# Local oxygen concentration reversal from hyperoxia to hypoxia monitored by optical-resolution photoacoustic microscopy in inflammation-resolution process

**DOI:** 10.1016/j.pacs.2025.100730

**Published:** 2025-05-17

**Authors:** Yizhou Tan, Min Zhang, Zhifeng Wu, Jingqin Chen, Yaguang Ren, Chengbo Liu, Ying Gu

**Affiliations:** a Department of Laser Medicine, the First Medical Center, Chinese PLA General Hospital, Beijing 100853, China; b Laser Medicine Center, Hainan Hospital, Chinese PLA General Hospital, Sanya 572013, China; c Research Center for Biomedical Optics and Molecular Imaging, Key Laboratory of Biomedical Imaging Science and System, Shenzhen Institute of Advanced Technology, Chinese Academy of Sciences, Shenzhen 518055, China

**Keywords:** Optical-resolution photoacoustic microscopy, Hyperoxia, Acute sterile inflammation, Gouty arthritis, Spontaneous resolution, Photobiomodulation

## Abstract

Current consensus suggests a simultaneous occurrence of hypoxia and inflammation. For the first time, we observed a hyperoxia state during the initiation stage of gouty arthritis (GA) via optical-resolution photoacoustic microscopy. GA as a paradigm of acute sterile inflammation, has been regarded as a single process. However, our experimental results demonstrated that the onset-resolution inflammation process composed of two sub-processes with different features. In the initial sub-process, inflammation and resolution events appear in hyperoxia state (1st-5th days). In the subsequent sub-process, post-resolution events appear in hypoxia state (6th-15th days), which is related with the second wave of immune response. Furthermore, we demonstrated that the inflammatory cytokines together with hyperoxia levels in initial sub-process can be downregulated by photobiomodulation, resulting in the hypoxia levels in subsequent sub-process were inhibited. Our results unveiled the detailed progress of GA and provided potential non-invasive monitoring and treatment strategies.

## 1 Introduction

Oxygen metabolism is essential for function and survival of most cells of the mammal body. Decreased oxygen concentration (hypoxia) is a prominent feature of pathological states encountered in bacterial infection, inflammation, wounds, cardiovascular defects and cancer, which arises when cellular oxygen demand exceeds supply[1]. In inflammation condition, hypoxia is a combined result of oxygen consumption by migrating inflammatory cells, local cell proliferation and the consumption of oxygen through activation of oxygenases that are expressed in multiple cell types in the inflammatory microenvironment[2], [3]. Studies have demonstrated that tissues within the microenvironment commonly experience concurrent hypoxia and inflammation[3], [4], [5], such as rheumatoid arthritis[6], [7], [8], [9], [10], [11] and post-traumatic osteoarthritis[12]. Emerging research suggests that chronic continuous hypoxia may have beneficial effects in autoimmunity diseases[13]. However, we observed an unconventional phenomenon that hyperoxia state (1st-5th days) turned into hypoxia state (6th-15th days) at same inflamed site in gouty arthritis (GA) for the first time.

GA is a paradigm of acute sterile inflammation[14], caused by deposited monosodium urate (MSU) crystals which intrigued an acute inflammatory response with severe pain, oedema and erythema in joints[15]. Then it normally resolved in a few weeks and might develops to chronic gouty arthritis and structural joint damage with persistent hyperuricaemia[16]. This condition affects over 55.8 million people worldwide, and are predicted to rise to 95.8 million in 2050[17]. Despite years of researches, the unclear pathological explanations of GA have hindered advancements in the diagnosis and treatment of this disease. For example, how does the cascade of immune reaction happen and why self-resolution occur even in the presence of urate crystals are still questions surrounding GA[18].

Although it has been known for centuries that the deposition of MSU crystals causes GA, it has only been two decades since the discovery of the activation of the NALP3 inflammasome, resulting in acute inflammation[19], [20]. While the self-limiting nature of GA typically leads to the return of inflammation cytokines to normal levels within a few weeks, recent studies have shown that resolution does not mark the end of inflammation[21]. A previously overlooked second wave of leukocyte influx into tissues has been observed post-resolution[22]. Oxygen metabolism, a key feature at the inflamed GA microenvironment, has not been reported before according to our knowledge.

We found that onset-resolution inflammation process composed of two sub-processes. In the initial sub-process, inflammation and resolution events appear in hyperoxia state. In the subsequent sub-process, hyperoxia reversed to hypoxia state. Furthermore, we found that local oxygen concentration levels in tissue were regulated by external light irradiation (627 nm). The inflammatory cytokines concentration together with hyperoxia levels in initial sub-process can be downregulated, resulting in the hypoxia levels in subsequent sub-process were inhibited.

Three experiments were carried out aiming at monitoring above-mentioned the unconventional hyperoxia and its reversal to hypoxia subsequently in acute sterile inflammation. Experiments were conducted as follows: (I) Temporal variation process of initiation stage of GA in 24 h. (II) Spontaneous resolution of GA acute inflammation in 15 days. (III) Regulation of hyperoxia/hypoxia levels by external light irradiation.

**Fig. 1:**
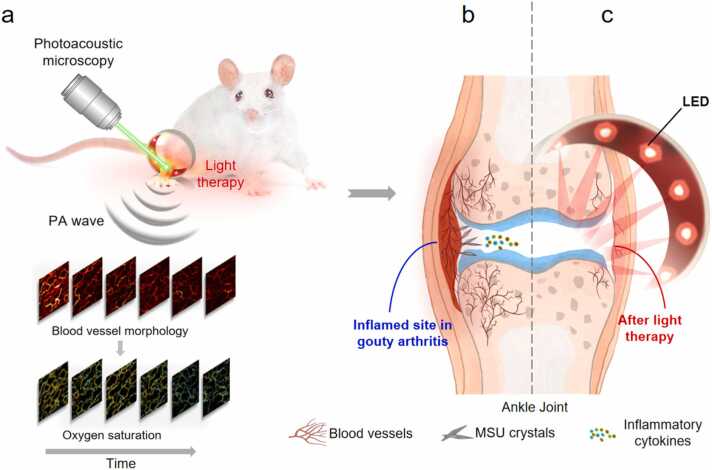
(a) Optical-resolution photoacoustic microscopy was used to monitor blood vessel morphology and oxygen saturation of SD rat ankle for 15 days. A ring-shape LED light source of 627 nm was used to irradiate rat ankle. (b) Acute inflammation was triggered by MSU crystal deposition with vasodilatation and inflammation cytokines accumulated. (c) An external light irradiation composed of LED alleviated the inflammation symptoms.

The acute inflammatory response of GA was mimicked by injection MSU suspension into ankles of Sprague-Dawley (SD) rats. We used a previously built dual-wavelength optical-resolution photoacoustic microscopy (OR-PAM) to monitor morphological changes of blood vessels label-freely[23]. Oxygen saturation level was extracted from the PAM signals with inﬂammatory cytokines tested by Enzyme-Linked Immunosorbent Assay (ELISA) and histology exam in various time points. Meanwhile, ultrasonography was applied to scan the joints with the MSU accumulation with swelling been ruled and pain threshold measure by von Frey test. Based on the observation, we further utilized external light irradiation to downregulate the inflammatory response and investigated the interactions of photobiomodulation and inflammation-resolution at different stages. The sketch of the study was illustrated in Fig. 1.

## 2 Materials and methods

### 2.1 Animals

Male SD rats, weighing (450 ± 50) g, aged 11 weeks, were used in this study. All animals were provided by Guangdong Medical Laboratory Animal Center. The rats were housed in a closed environment with temperature control (24 ± 2℃) and 12 h light/dark cycle, drinking sterile water, and eating standard pellet feed AD libitum. Animal experiments were conducted in accordance with the Institutional Animal Care and Use Committee of Shenzhen Institute of Advanced Technology (SIAT-IACACC-240111-YGS-CJQ-A2453).

### 2.2 Gouty arthritis model

After three days of adaptive feeding, 0.25 mL of MSU (Sigma-Aldrich) suspension (5 g/100 mL) was injected into the ankle joint cavity at an angle of 45° to the tibia from the lateral side of the right ankle joint. Contralateral bulging of the joint capsule was considered as a successful injection. The rats were randomly divided into two groups, which are gouty arthritis group (GA group) and low-level light therapy group (GA+LT group). All protocols adhered to the NIH standards for the care and use of laboratory animals and followed the ARRIVE guidelines for reporting experiments.

### 2.3 Swelling index (SI) measurement

The formation of ankle oedema is one of the main symptoms of acute GA symptoms[24], [25], [26]. To assess the inflammation response of the ankle, the circumference of the ankle was measured by a flexible non-stretch thread around the joint along a horizontal line and the length of the thread was read by a digital caliper. Three consecutive measurements were performed per ankle, and the average circumference was recorded. The swelling index was then calculated using the formula:

(1) $$\mathrm{SI}=\frac{S_{n}-S_{0}}{S_{0}}\times 100\%$$

where$S_{0}$is the ankle circumference before the injection of suspension, $S_{n}$ is the ankle circumference at day n after since the injection.

### 2.4 Von Frey test

To assess mechanical allodynia and tactile sensitivity of the rat’s ankle, von Frey filament test was performed using a series of calibrated monofilaments (Stoelting, USA) applied to the plantar surface of the hind paw[27], [28]. After acclimating for half an hour, the von Frey test probe was used to slowly and gently stimulate the middle plantaris of the measured hind limb of the rat. The probe was bent for a few seconds to observe the foot contraction response of the rat. If the rat showed a rapid foot contraction response due to the stimulus, it was marked as a positive response, and if the foot contraction response was caused by physical activity, it was not counted. The rats were tested with filaments of each threshold 10 times, and if there were at least 6 positive reactions out of 10 times, the corresponding threshold of filaments was the paw withdrawal threshold (PWT) of the rats. Otherwise, the test continued with the next higher dose of filaments until the rats showed at least six positive responses to filaments at a certain threshold. The interval between the two adjacent tests was set to 1 min to allow the rats to fully recover from the previous stimulus[29].

### 2.5 Light irradiation device and the treatment plan

The light irradiation device used in this experiment was made by a LED ring. The device was a cylindrical structure with a diameter of 5 cm and a width of 2 cm, with 10 LED beads installed equidistantly inside. The peak wavelength of the LED is 627 nm, the power density is 10 mW/cm^2^, the full width half maximum is 6 nm, measured by spectral power meter (SENSINGM, China).

Before each light irradiation treatment, the hair on GA ankle joint of the rats were removed to ensure that there was no hair in the illuminated area. The GA+LT group started treatment on the day of MSU injection and continued treatment for 15 days for 1 h per day. Isoflurane was used to anesthetize rats during the light irradiation treatment. Before the treatment, a spectral power meter was used to measure and calibrate the power density of the light irradiation device.

### 2.6 Ultrasonography (US) scan and assessment

US scans were performed using a Vevo®2100 device (Esaote Biomedica, Genoa, Italy) equipped with a high-frequency (13–24 MHz) linear array transducer. All US examinations were performed by two experienced sonographers, who were blinded to the injection type at all time points. Before the study, the sonographers reached consensus on the scanning technique to adopt and the gout-related US findings to evaluate. Rats were scanned using a multiplanar technique. The inclination angle was adjusted in order for it to be perpendicular to the joint surface. B-mode gain was initially set in order to obtain maximal contrast among tissues and was successively reduced to its lowest level, allowing only visualization of hyperechoic structures, using the bony cortex as reference.

The following US features of MSU crystal deposition were assessed: bright stippled aggregates (BSA), as it was previously described as among the most frequently identified elementary lesions of gout. BSA is defined as intra-articular heterogeneous hyperechoic foci with or without posterior shadowing over a hypo- or anechogenic background. Finally, all US images were interpreted in conjunction with a third, blinded, experienced sonographer. Discrepancies were resolved by consensus among the three sonographers.

### 2.7 Optical-resolution photoacoustic microscopy (OR-PAM) imaging system

**Fig. 2:**
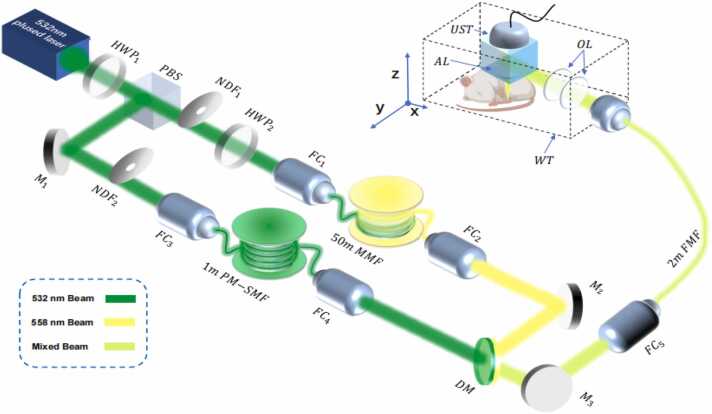
**Optical path diagram of OR-PAM system.** HWF: half-wave plate, NDF: neutral density filters, PBS: polarizing beam splitter, M: mirror; FC: fiber coupler, DM: dichroic mirror, MMF: graded-index multimode fiber, PM-SMF: polarization-maintaining single-mode fiber, FMF: few-mode optical fiber, OL: optical lens, UST: ultrasonic transducer, AL: acoustic lens, WT: water tank.

An OR-PAM system with dual-wavelength pulsed laser was developed and sketched in Fig. 2. The system used a tunable diode pump laser system (SPOT-10–200–532, Elforlight) with a repetition frequency of 1 kHz to provide pulse laser with a wavelength of 532 nm. The laser beam passing through a high-power polarizing beam splitter prism (HPBS0325–532, Hengyang Optics) was divided into two beams, one beam was coupled to a 50 m multi-mode optical fiber (GIMMSC(50/125)CHT, Fibercore) through a fiber coupler (CFC11P-A, Thorlabs) to generate a laser with a wavelength of 558 nm through stimulated Raman scattering. A zero order waveplate air spaced (WPZ-532-λ/2, Hengyang Optics) was placed before the fiber coupler to adjust the incident polarization state so that the SRS efficiency in the fiber is maximized. The other beam was coupled into a 1 m polarization-maintaining single-mode fiber (PM-S405-XP, NUFERN) for collimating the laser beam. Given that the group velocity of light in optical fiber is approximately 2.0 × 10⁸ m/s, the calculated propagation delays were ∼5 ns for the 532 nm pulse and ∼250 ns for the 558 nm pulse. This resulted in an effective inter-pulse delay of approximately 245 ns. This delay was sufficient to achieve complete temporal separation of the two photoacoustic signals within each A-line. It effectively avoided signal overlap or interference while ensuring that the Raman process had adequate time to produce a high-energy 558 nm pulse. The two laser beams were combined together through a dichroic mirror (FF535-SDi01–25x36, Semrock) and coupled into a 2 m few-mode optical fiber (SM1250SC(9/125)CP, Fibercore). The laser beam passed through a photoacoustic combiner composed of a right-angle prism and a rhombus prism and is focused on the sample surface. The generated PAM signal was reflected by a thin layer of silicone oil between two prisms and then detected by an ultrasonic transducer (V214-BB-RM, OlympusNDT) with a center frequency of 50 MHz and a bandwidth of 100 %. At the end of the photoacoustic combiner, an acoustic lens with an NA of 0.55 was installed. The photoacoustic combiner was immersed in the water tank and the z-position of the photoacoustic combiner is adjusted to maximize sensitivity. The laser beam and ultrasound transducer were mounted on a scanning stage (VT80, PI) and moved mechanically in 5 μm increments in the scanning direction (i.e., along the x and y axes). The detected PAM signals were pre-amplified by a commercial amplifier (5073R, Olympus) to the photoacoustic signal detected by the sensor, and digitally processed by a 200-MS/s data acquisition card (CS1422, GaGe).

System synchronization was managed by a centralized FPGA-based controller, which coordinates the laser source, the data acquisition card, and the scanning motor. Upon receiving the scan initiation signal, the FPGA issues synchronized TTL pulses with a common rising edge to trigger laser firing, acquisition start, and motor movement. This ensured that every laser pulse, A-line acquisition, and scan step are temporally aligned. The data acquisition card was configured with a programmable delay to precisely align the sampling window with the expected arrival time of both 532 nm and 558 nm signals. The system utilized a data acquisition rate of 200 MS/s, which corresponds to a temporal resolution of 5 ns per sample. This sampling density was adequate to temporally resolve the two signals and avoid any misclassification during sO₂ estimation. According to the Nyquist criterion, our 50 MHz center-frequency transducer requires 100 MS/s for accurate reconstruction, therefore the 200 MS/s sampling rate provided a robust margin to ensure fidelity and prevent aliasing. The ankle joint was marked to ensure that each image was in the same area, shown in Fig. S1.

### 2.8 Calculation of vascular structural parameters

For blood vessels, the diameters of the vessels can be determined by calculating the Euclidean distance between the two points where the tangent lines intersect with the blood vessel edge and the cross-section line. This is feasible because the tangent lines at each point on the center line are perpendicular to the blood vessel cross-section. Vascular density (VD) is determined by quantifying the proportion of the vascular lumen in the Z-axis maximum amplitude projection (MAP) image after binary reconstruction. Its numerical value ranges from 0 to 1. Vessel tortuosity was assessed by three complementary metrics: (1) The distance metric (DM), calculated as the ratio of a vessel segment's actual path length to its linear end-to-end distance; (2) The inflection count metric (ICM), derived by multiplying DM with the number of curvature sign changes along the vessel trajectory; and (3) The sum-of-angles metric (SOAM), defined as the cumulative curvature magnitude at all discrete points along the three-dimensional centerline divided by the total path length.

### 2.9 Quantification of blood oxygen saturation

A mask was firstly generated from the 532 nm photoacoustic image using intensity-based thresholding and normalization. It selectively includes regions with high photoacoustic signal intensity, corresponding to blood vessels, and excludes irrelevant or low-signal regions such as surrounding tissue and noise-prone background pixels. The representative local blood oxygen saturation (sO_2_) values were obtained by calculating the mean sO_2_ within this masked region for each animal and each time point.

To quantify the (sO₂) at inflamed joints, we employed the dual-wavelength OR-PAM system with excitation wavelengths at 532 nm and 558 nm, operating at a pulse repetition rate of 1 kHz. The selection of these two wavelengths is based on their differential absorption by oxy-hemoglobin (HbO₂) and deoxy-hemoglobin (HbR): 532 nm lies near an isosbestic point (equal absorption of HbO₂ and HbR), while 558 nm exhibits stronger absorption by HbR than HbO₂. This spectral contrast enables separation of the contributions from HbO₂ and HbR in the detected photoacoustic signals.

The photoacoustic intensity *I*_PA_ at each wavelength is related to the optical absorption coefficient *μ*_*a*_*,* which depends on the concentration of HbO₂ and HbR:

(2) $$I_{\mathrm{PA}}=\Gamma \eta F\mu_{a}=\Gamma \eta FC_{\mathrm{Hb}}\left[\varepsilon_{\mathrm{Hb}O_{2}}sO_{2}+\varepsilon_{\mathrm{HbR}}(1-sO_{2})\right]$$

where Γ is the local Griineisen coefficient, η is the optical-to-acoustic conversion efficiency, *F* is the local optical fluence, $\mu_{a}$is the absorption coefficient, $C_{\mathrm{Hb}}$ is the total hemoglobin concentration, $\varepsilon_{\mathrm{Hb}O_{2}}$and $\varepsilon_{\mathrm{HbR}}$are the molar extinction coefficients of HbO_2_ and HbR at a given wavelength.

After acquisition, the raw PA signals were first processed via Hilbert transformation to extract their amplitude envelopes. These were then subjected to Z-axis maximum amplitude projection to produce two-dimensional representations of vascular absorption at each wavelength. By acquiring pixel-wise photoacoustic signals at both wavelengths and applying a linear spectral unmixing algorithm, we calculated the local sO_2_ value for each vascular pixel, and average sO_2_ values in regions of interest were computed to monitor temporal changes during disease progression and phototherapy. This method has been widely adopted in functional OR-PAM systems and provides reliable, label-free quantification of tissue oxygenation at microscopic resolution[27]. In our system, the imaging the optical illumination from both wavelengths shared the same optical path and focal geometry. We assumed a spatially uniform and wavelength-independent fluence distribution within the imaging region. As a result, the relative differences in PA signal amplitude between the two wavelengths predominantly reflected hemoglobin absorption contrast rather than fluence variations. Therefore, no explicit fluence calibration or correction was applied in this study.

To validate the accuracy of our sO_2_ measurements, we imaged dorsal skin of nude mice, with result shown in Fig. S2a, b. The arteries were clearly visualized in red hues corresponding to high sO2 levels (>90 %), while veins appeared in yellow-green colors with moderate sO_2_ values (∼65 %). By changing the image location, we found sO_2_ value constant. These results were consistent with the expected physiological range of sO_2_ in arterial and venous blood, as documented in the current literature[28], [29].

We further carried out repeatability tests on our OR-PAM system by conducting two consecutive sO_2_ measurements at the same location. The blood vessel morphology and sO_2_ images were presented in Fig. S2c, d. Using the calculation method employed in our study, we obtained a sO_2_ value of 63.92 % in the first scan (Fig. S2c) and 62.31 % in the second scan (Fig. S2d). The results indicated a consistent sO_2_ evaluation under identical physiological and environmental conditions.

### 2.10 Enzyme-Linked Immunosorbent Assay (ELISA)

Under isoflurane anesthesia, blood samples of rats were obtained via tail-tip with volume controlled. In Experiment I, blood samples were collected at one day before the experiment (pre-injection, designated as 0 h), 12 h, and 24 h post intra-articular MSU or saline solvent injection. Experiments II-III involved longitudinal sampling in GA and GA+Light groups at one day before the experiment (pre-injection, designated as 0 h), 12 h, and 1, 2, 3, 5, 7, 11, 15 d. Samples were clotted at room temperature (1 h), then centrifuged at 10,000 rpm (15 min, 4°C) for serum isolation. Aliquots were stored at −80°C until analysis. Serum IL-1β, IL-6, and TNF-α levels were quantified using manufacturer-validated ELISA kits. In order to minimize potential influence on PAM imaging results, blood collection was conducted after each imaging session.

### 2.11 Histological Analysis

The rat ankle joint tissues of rat were fixed in 4 % paraformaldehyde for 48 h, decalcified in EDTA solution for approximately three months, embedded in paraffin wax, and cut into 4 μm thick slices, and then, ankle joint slices were stained with hematoxylin-eosin (H&E). The histological sections were later stained for observation under the light microscope.

### 2.12 Statistical analysis

Data were analyzed with GraphPad Prism 7.0 software (GraphPad Software, La Jolla, CA, USA) and expressed as mean ± SD. Analysis of Variance (ANOVA) was used to determine significant differences among rat groups. *P* values < 0.05 were considered as statistically significant.

## 3 Results

### 3.1 Temporal variation process of initiation stage of GA process in 24 h

**Fig. 3:**
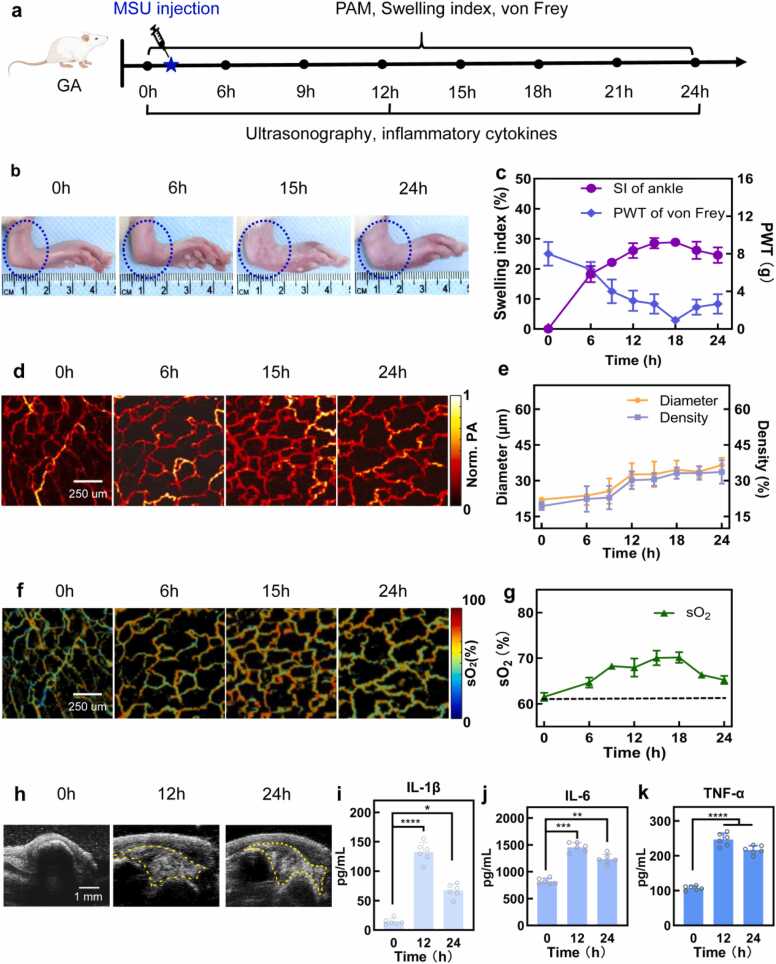
**Observation of GA development in SD rats in 24 h.** (a) Experimental schedule of monitoring. (b) Photos of rat ankle before and after MSU injection. (c) Swelling index and paw withdrawal threshold (PWT) of Von frey of GA ankle in 24 h. d) PA images of ankle blood vessels within 24 h. (e) Diameter and density of blood vessels in GA ankles. (f) sO_2_ images of ankle blood vessels within 24 h. (g) sO_2_ level of ankle blood vessels within 24 h with dashed line indicating the normoxia level. (h) Ultrasonography scan of GA ankles at various time points with dashed line indicating MSU area. (i-k) Concentration of inﬂammatory cytokines in blood serum: IL-1β, IL-6 and TNF-α respectively. Animal number n = 6, mean ± SD; *p < 0.05, * *p < 0.01, * **p < 0.001, * ** *p < 0.0001.

Acute GA was induced in SD rat by injecting MSU suspension into the ankle joint at the beginning of the experiment. An in-house OR-PAM system was utilized to image the injected ankle every 3 h from 6 to 24 h post-injection. Meanwhile, the swelling index, von Frey test of the injected ankle, ultrasonography scan and ELISA tests for inflammatory cytokines were performed subsequently. The experimental protocol is outlined in Fig. 3a. Significant oedema was observed in the ankle injected with MSU suspension 6 h post-injection, worsening by 24 h, as recorded in photos in Fig. 3b. To quantify the oedema, the swelling index was calculated based on changes in ankle circumference. The ankle circumference showed a gradual increase, peaking at 28 % at 18 h. At 24 h, the swelling index remained 24 % higher than the baseline value, as illustrated by circles in Fig. 3c. In contrast, a control group injected with an equal volume of saline showed that the swelling index returned to normal levels in 6 h, as shown in Supplementary Fig. S3a. The reduced paw withdrawal threshold (PWT) in response to the von Frey filament test indicated heightened pain sensitivity in the ankle due to the activation of inflammation. PWT of the GA joint decreased from 8 g to 6 g in 6 h, and further declined to 0.9 g by 18 h, as illustrated by diamonds in Fig. 3c. PWT of the control group kept constant for 24 h, results in Fig. S3b. Severe oedema and pain were most common symptom experienced by GA patients and used as clinical diagnosis criteria. However, both oedema and pain feeling differenced individually and therefore cannot be used to quantify the progress of the disease.

Aiming at finding the relationship between blood vessel morphology and oxygen saturation with acute inflammation, we conducted PAM scan on the GA joints. The average blood vessel diameter and density were quantified from the captured PAM images, as illustrated in Fig. 3d, with the statistical results plotted in Fig. 3e. Both the diameter and density remained stable during the initial 9 h of the MSU injection, then abruptly doubled between 9 and 12 h, followed by gentle fluctuations. Oxygen saturation (sO_2_) of the blood vessels was derived from the PAM signals in Fig. 3f and presented in Fig. 3g. Different to the relevant stable vasculature morphology, the average sO_2_ in the measured area increased from 63 % to 68 % within 9 h, subsequently reaching 71 % at 18 h before declining to 65 % at 24 h. We found the rapid elevation in blood flow and oxygen levels correlated with changes in ankle conditions accurately, including oedema and pain thresholds which all peaked at 18 h. It indicated at the early stage of the GA infection, sO_2_ level is more sensitive to the inflammation condition than vasculature morphology.

The correlation between sO_2_ level and inflammation was further demonstrated by the concentration of inflammatory cytokines, including IL-1β, TNF-α, and IL-6. All of three measured inflammatory cytokines exhibited an increase at 12 h post-MSU injection before slightly decreasing at 24 h, as shown in Fig. 3i-k. Specifically, the concentration of IL-1β, a key trigger of innate immune responses, increased significantly by eightfold at 12 h, indicating a robust inflammatory reaction. By contract, inflammatory cytokines in the control group kept stable for 24 h, with results shown in Fig. S3c-e. Furthermore, analysis of the ultrasonography image in Fig. 3h revealed the accumulation of MSU suspension in the joint cavity at 12 h post-injection, and persisting at 24 h.

### 3.2 Spontaneous resolution of acute GA inflammation in 15 days

**Fig. 4:**
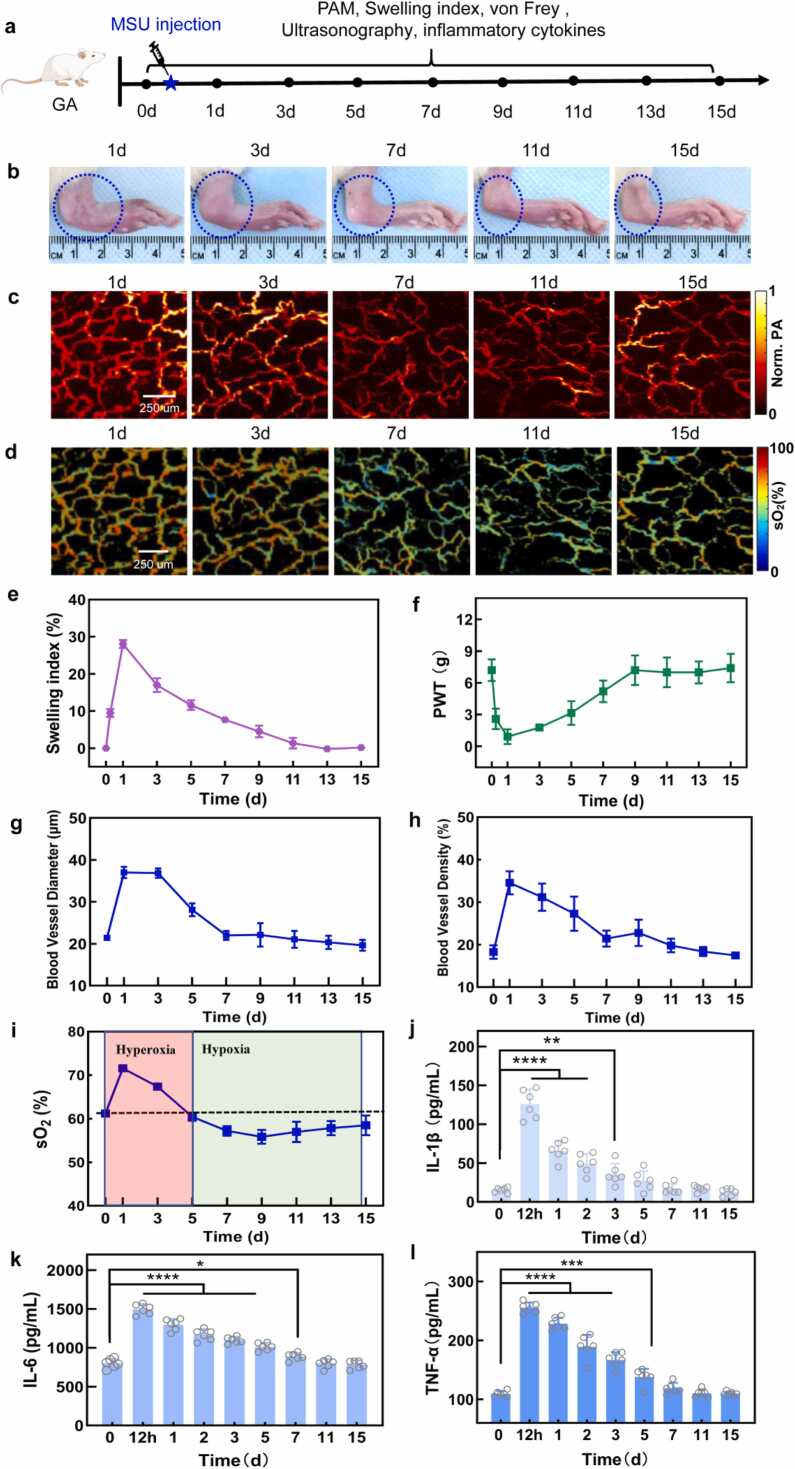
**Observation of GA inflammation-resolution in rats in 15 days.** (a) Experimental schedule of the observation. (b) Photos of rat ankle. (c) OR-PAM images of morphology of ankle blood vessels. (d) sO_2_ images of ankle blood vessels. (e) Swelling index of GA ankle. (f) PWT value of von Frey filament test. (g) Averaged diameter of blood vessels in GA ankles. (h) Averaged density of blood vessels in GA ankles. (i) Averaged s sO_2_ level of ankle blood vessels with dashed line indicating the normoxia level. (j-l) Concentration of inﬂammatory cytokines: IL-1β, IL-6 and TNF-α. Animal number n = 6, mean ± SD; *p < 0.05, * *p < 0.01, * **p < 0.001, * ** *p < 0.0001.

The acute inflammation normally self-limited and settled down in 2 weeks clinically, then progress into chronic inflammation[19], [30]. Therefore, we extended the observation of the GA rat model to 15 days with PAM, swelling index, von Frey, ultrasonography and inflammatory cytokines measured. The experiment was illustrated in Fig. 4a with joint photos in Fig. 4b. The vasculature morphology was plotted in Fig. 4c and statistical result in Fig. 4g. The average blood vessel diameter and density of the GA joints increased double-folded at 24 h then gradually decreased to a normal average at day 7. The sO_2_ of blood vessels was extracted from the PAM images (Fig. 4d) and averaged values are presented in Fig. 4i. The sO_2_ of GA group experienced a sharp increase to 72 % on day 1, followed by a gradual decline to 59 % by day 5. However, sO_2_ further decreased to 56 % by day 9 and kept below the baseline until day 15, showing a constant hypoxia state in the joint locally. A hypoxia state was connected to chronic inflammation in GA progress. Histopathological results are available in Supplementary Fig. S4 which showing a large number of inflammatory cell infiltration. According to our knowledge, this is the first in vivo observation of GA development from hyperoxia to hypoxia.

The results of swelling index and PWT measurements were consistent with the vasculature morphology which peaked at day 1 and gradually decreased to the normal level by day 9. Meanwhile, the inflammatory cytokines, including IL-1β, TNF-α, and IL-6, were quantified using ELISA kits from 12 h to 15 days, with concentration results shown in Fig. 4j-l, respectively. The peak concentrations of all three inflammatory cytokines were recorded at 12 h post-treatment, gradually decreasing over time to normal level around day 7. However, after day 7 the chronic inflammation appeared as demonstrated by histological findings in the Supplementary Fig. S4. A large number of inflammatory cells were infiltrated in pathological section. Although the local chronic inflammation was not found in vasculature morphology and peripherical blood test, the sO_2_ value showed clear hypoxia since day 7, which is evidence for chronic inflammation. By comparison, a control group injected with an equal volume of saline showed no fluctuation in swelling index, PWT and inflammatory cytokines in Fig. S5. Overall, we demonstrated sO_2_ value of vasculature system near joint is a sensitive bio-marker for inflammation monitoring.

### 3.3 Regulation of hyperoxia/hypoxia levels by external light irradiation

**Fig. 5:**
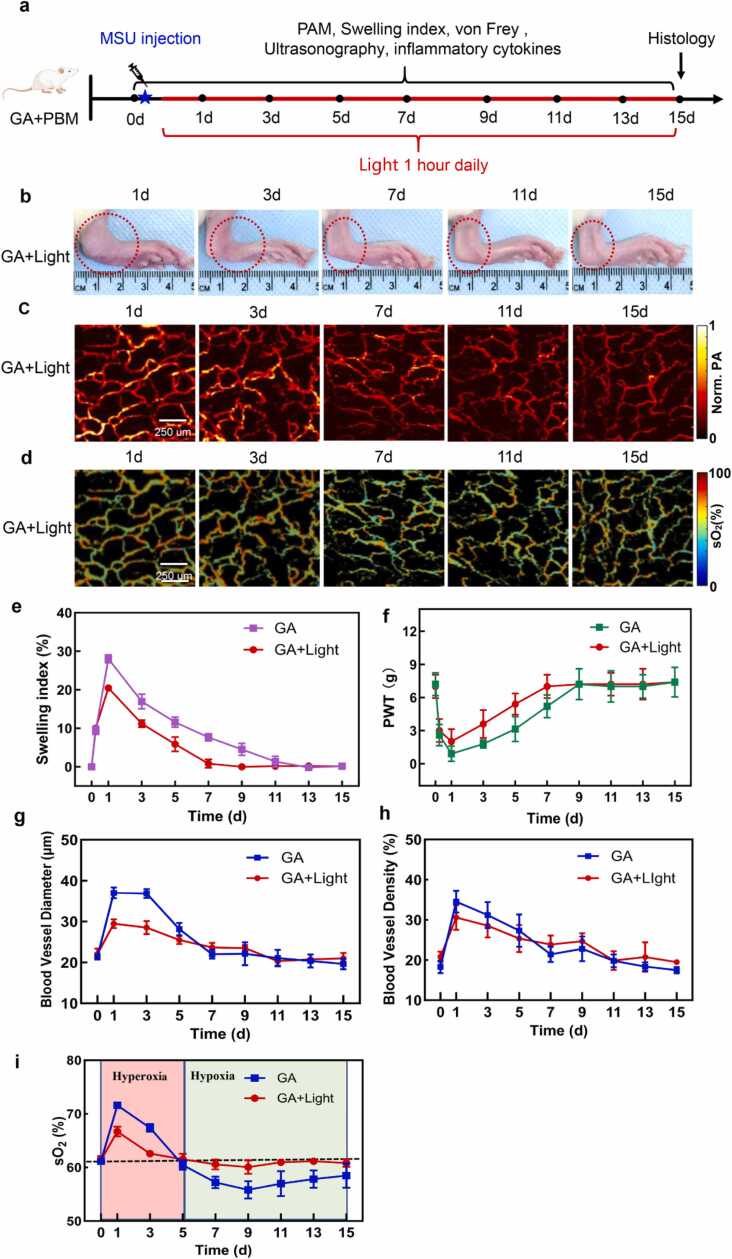
**Effect of external light irradiation on GA rats in 15 days.** (a) Experimental schedule of the external light irradiation. (b) Photos of rat ankle in GA+Light group. (c) OR-PAM images of morphology of ankle blood vessels. (d) sO_2_ images of ankle blood vessels. (e) Ankle swelling index of GA rats. (f) PWT value of von Frey filament test. (g) Comparison of vessel diameter changes between GA and GA+Light group. (h) Comparison of changes in vascular density between GA and GA+Light group. (i) Comparison of blood oxygen saturation between GA and GA+Light group, showing a hyperoxia and hypoxia sub-processes, with dashed line indicating the normoxia level. The statistical analysis of e-i is presented in Tab. S1–5. Animal number n = 6, mean ± SD.

We developed a ring-shaped LED-based light system to treat GA rats for 1 h daily over a period of 15 days. The device emitted 627 nm light with a power density of 10 mW/cm^2^ at the central point of the ring. The treatment schedule is illustrated in Fig. 5a and device shown in Fig. S6. Temperature of joint surface during the external light irradiation was shown in Fig. S7. To assess the impact of external light irradiation on acute inflammation in GA, we performed PAM imaging before the injection and post-treatment every two days from day 1 to day 15. Analysis of the structural images of blood vessels revealed that hemangiectasis in GA+Light group was less severe than the GA group on day 1, after the first light irradiation. As illustrated in Fig. 5g and h, the average diameter of blood vessels in the GA+Light group was 27 % narrower than the GA group after the onset of GA, and the density of blood vessels in the GA+Light group was 13 % less dense than the GA group after the onset of the inflammation. Then both group experienced vasculature structure recovered to normal value around day 7.

The oxygen saturation level sO_2_ of blood vessels was extracted from the PAM images (Fig. 5d) and averaged values are presented in Fig. 5i. The GA group experienced a sharp increase to 72 % on day 1 showing hyperoxia, followed by a constant hypoxia state until the end of the experiment. In contrast, the GA+Light group showed less variation in sO_2_ levels, with the highest value of 67 % on day 1 and fluctuating around 60 % since day 5, equivalent to the normoxic sO_2_ value of 61 % on day 0 before the injection. It kept consist until the end of the experiment without experiencing a hypoxia stage.

**Fig. 6:**
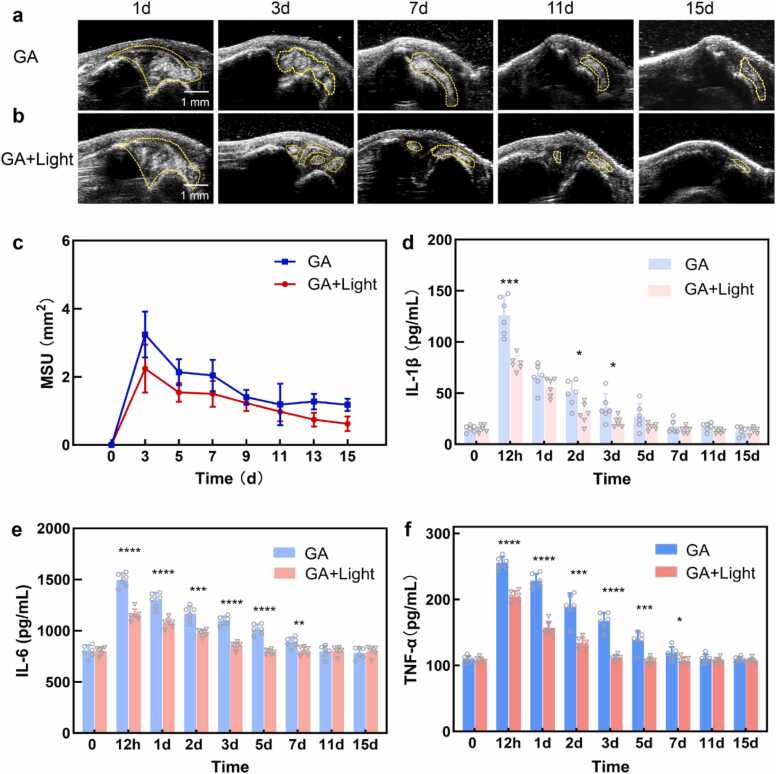
Effect of external light irradiation on GA rats in 15 days. (a, b) Ultrasonography scan of GA and GA+Light group over 15 days. Yellow dashed lines represent MSU accumulation. (c) MSU area of GA and GA+Light group in 15 days. (d-f) Analysis of serum inflammatory cytokines IL-1β, IL-6 and TNF-α at various time points. Animal number n = 6, mean ± SD; *p < 0.05, * *p < 0.01, * **p < 0.001, * ** *p < 0.0001.

As shown in Fig. 5e and 5f, both swelling index and mechanical pain sensitivity (PWT) were elevated in the GA group and progressively improved in the GA+Light group following treatment. An additional data point at 4 h post-injection was included before the initiation of light therapy to confirm that there was no significant difference between the two groups during the onset of the acute inflammation. By comparison of the swelling index and PWT value, effect of photobiomodulation showed on day 1 after the first external light irradiation. The external light irradiation alleviated the swelling and pain, rendering the value in GA+Light group returning to normal level 4 and 2 days earlier than the naturally recovering GA group. Results were shown in Fig. 5e and f respectively. Meanwhile, a commercial animal ultrasonography was utilized to scan the GA ankle every two days over a 15-day period, with ultrasonography images depicted in Fig. 6a. On day 1, bright stippled aggregates indicative of MSU accumulation were identified in both the GA and GA+Light groups, as denoted by dashed yellow lines. Subsequently, the MSU suspension in the GA+Light group gradually dissipated, with no high echogenicity observed in the joint cavity by day 15. In contrast, MSU presence persisted in the GA group until the end of the observation period. The area of the MSU region was quantified and illustrated in Fig. 6c for both groups.

Inflammatory cytokines, including IL-1β, IL-6 and TNF-α, were quantified using ELISA kits from 12 h to 15 days, with concentration results shown in Fig. 6d-f, respectively. The peak concentrations of all three inflammatory cytokines were recorded at 12 h post-treatment, gradually decreasing over time. Notably, the GA+Light group exhibited lower concentrations in all tests, indicating the therapeutic efficacy from the initial treatment session. The change of Inflammatory cytokines correlated with the vasculature morphology which showed early recover after light irradiation. The result is further supported in histopathological results of synovium of GA+Light group shown in Fig. S4. Compared with GA group, the infiltration of inflammatory cells into synovial tissue in GA+Light group was significantly less from the pathological section.

## 4 Discussion

The inflammation and immunity response can vary depending on the oxygen level deviated from the normal value in microenvironment[2], [31]. While hypoxia, where cellular oxygen demand surpasses supply, is a prevalent occurrence in inflammatory processes such as rheumatoid arthritis[6], [7], [8], [9], [10], [11], we observed a unique scenario in this work where hyperoxia and inflammation coexist at the inflamed site.

Three experiments were carried out to monitoring the dynamic immune reaction of GA in the same animal via OR-PAM. In experiment I, we unveiled a hyperoxia state during the onset stage of acute GA inflammation. According to our knowledge, this is the first report of hyperoxia state during the activation of immune response in GA. In Experiment II, we tracked the longitudinal dynamics of spontaneous remission over 15 days, and we noted an unconventional occurrence where the hyperoxia state (1st-5th days) transitions to a hypoxia state (6th-15th days) at the same inflamed site. In Experiment III, an external light irradiation is introduced as external mediator of resolution via photobiomodulation principle. We found that immune reaction in second sub-process can be suppressed by downgrading the blood oxygen levels in the first sub-process. Measurement results collected by consecutive PAM were further demonstrated by ultrasonography imaging, pain threshold assessments, swelling index of ankle, measurements of inflammatory cytokine levels, and tissue section analysis.

In experiment I, we found that hyperoxia state and inflammation occurred concurrently at inflamed site of GA ankles. The representative value of normoxia and hyperoxia are around 61 % and 72 % respectively in Fig. 3g. It is known that the MSU crystals triggered the leukocytes recruitment and their accumulation, release of active interleukin IL-1β for initiating inflammatory responses, also known as ‘first wave of immune response’[32], [33]. A potential explanation for hyperoxia state could be the microvascular events caused by the first wave of immune response. The changes in microvascular form and function lead to vasodilatation and made the blood vessels more permeable with increased blood flow to the inflamed site, and allowing mediators for inflammation to enter in interstitial space[34], [35]. Thus, microvascular oxygen supply outstrips cellular oxygen demand.

Acute gout is an auto-inflammatory disease characterized by self-limiting inflammation[15], [33]. Without therapy, a gout flare usually resolves within one or two weeks[18], [36]. In experiment II, spontaneous remission process was studied by tracking longitudinal dynamics of oxygen saturation and blood vessel morphology. We found that hyperoxia state (in 1st– 5th days) turn into hypoxia state (6th-15th days) at same inflamed site, referred to as ‘reversal of oxygen level’.

As Fig. 4i shown, two immune response events appear sequentially: the former in hyperoxia state (lasted for about 5 days) associated with the first wave of immune response, and the latter in a hypoxic state (for about 10 days) associated with the second wave of immune response. This ‘reversal of oxygen level’ phenomenon is experimental evidence for the second wave of immune response that occurs in the sub-process (6th-15th days). In Fig. 4i, the left part of blue curve had its peak value closed to 72 %. In terms of theory, the inflammation-resolution process is complete, typically last for 5 days, because the normoxic state appears briefly while the turning point take a value 60 % at 5th day. The right part of blue curve demonstrates the existence of the second wave of immune response. Its pathological model can be explained by the theory that resolution is not the end of innate mediated immune responses to infection or injury[19]. There are further immunological activities occurring after the resolution cascade is complete. Accordingly, in this study, the further immunological activity after the 5th day is a reasonable cause of reversal of oxygen level phenomenon. The hypoxia state after the 5th day is attributed to the increased oxygen consumption of immune response, leading to cellular oxygen demand outstrips supply in microenvironment. This inflammatory hypoxia arises from a confluence of factors including the recruitment of inflammatory cells (such as neutrophils and monocytes), local proliferation of various cell types, and activation of oxygen-consuming enzymes[37], [38].

To the best of our knowledge, this is the first report detailing an initial hyperoxia process followed by a reversal of oxygen levels, leading into a subsequent hypoxia process in GA. For centuries, MSU crystals have been identified as the primary cause of GA, and the development of gout involves multiple stages[39]. It has only been two decades since the discovery of the activation of the NALP3 inflammasome, resulting in the production of active IL-1β and other inflammatory cytokines[14], [33]. The onset-resolution process has been regarded as a single process. For example, IL-1β raised sharply during the onset then decreased gradually during resolution, as shown in Fig. 4j. While the self-limiting nature of GA typically sees inflammation cytokines returning to normal levels within a few weeks, recent research challenges the notion that symptom resolution marks the end of inflammation[21]. Current studies suggest that resolution does not signify the conclusion of innate-mediated immune responses, with ongoing immunological activity observed post-resolution[21]. Literature[22] has unveiled a previously overlooked second wave of leukocyte influx into tissues that persists for weeks during acute inflammation. Our identification of hyperoxia and the subsequent reversal of oxygen levels align with the resolution and post-resolution phases of autoinflammation processes, offering valuable insights into the microenvironment at inflamed sites. However, while the concentration of inflammatory cytokines in the blood rose in parallel with hyperoxia, indicating a robust immune response, we recognized that the precise cause of this hyperoxia has not been elucidated in our current work. Additional metabolic analyses were critical to disentangle the contributions of immune cell activity. Potential assays included measurements of ROS production, mitochondrial oxygen consumption rate, mitochondrial membrane potential, and ATP production.

The experiment III was designed in which the inflamed ankle was irradiated by an annular array of light-emitting diode (wavelength at 627 nm). This external light source induced an anti-inflammatory effect[40], [41], [42] in inflamed tissue that is equivalent to endogenous ‘mediators of resolution’ of inflammation[43]. Results of experiment III indicate：Firstly, the downregulation of IL-1β and IL-6 and TNF-α levels correlates with light irradiation. Secondly, the inflammatory cytokines concentration together with hyperoxia levels can be downregulated in initial sub-process. For example, the peak value reduces from 72 % (bule curve) to 67 % (red curve) in Fig. 5i. Thirdly, the hypoxia levels in subsequent sub-process were inhibited by external light irradiation. As the right half part of Fig. 5i shown, the experiment value of blood oxygen levels (red curve) approached normal value in healthy tissue. The external light irradiation of 627 nm has significant influence to oxygen levels within biological tissues.

In Fig. 5i, GA group experienced a common hypoxia state after 5th day. However, the situation in GA+Light group was close to normoxic level after 5 days. The observation that hyperoxia levels decrease due to photobiomodulation while the duration remains constant provides further evidence for two waves of immune response that occur sequentially in two sub-processes with different pathological features. Our findings of PBM downregulating inflammatory cytokines and preventing hypoxia indicated an improvement in microenvironment with less damage observed, as demonstrated by the HE pathological section in Fig. S4. Previous research has shown that PBM can promote the phenotypic polarization of macrophages from the pro-inflammatory M1 phenotype to the anti-inflammatory M2 phenotype in conditions such as arthritis. This shift is crucial for tissue repair and the resolution of chronic inflammation. It is important to note that this phenotype shift does not compromise host defense or cellular immunity. Instead, it helps to control overactive inflammatory responses, which is beneficial for the overall immune function[42], [44]. Regarding neutrophils, PBM has been shown to reduce joint inflammation by inhibiting neutrophil viability and proliferation and decreasing the release of pro-inflammatory cytokines[42]. However, this does not necessarily mean that PBM causes immunosuppression. Neutrophils play a dual role in the immune response. While they are essential for the initial defense against pathogens, excessive activation can lead to tissue damage and chronic inflammation. By modulating neutrophil activity, PBM might be optimizing the immune response rather than suppressing it.

The findings reported in our experiments have two potential applications. Firstly, our experiments reveal two pathological phenomena, including hyperoxia state and two waves of immune response, which were hidden in inflammation-resolution process.

Our results introduce new insight for understanding the hyperoxia state that is considered a favorable factor for the spontaneous resolution. Resolution of acute inflammation was previously recognized as a single process dominated by a natural decay of pro-inflammatory signals. It is now clear that resolution is not a single process, but rather two sub-processes happened in sequence.

It should be noted that latter sub-process was previously overlooked in the earlier research. One possible reason is as follows: the pro-inflammatory response is stronger during the former sub-process, but the second wave of immune response is relatively weak during latter sub-process. Therefore, the biological effects produced by second wave of immune response was overwhelmed by the delay influence resulted in the former sub-process[45], [46].

Different types of biomarkers have been used for the early detection of gout or distinguish asymptomatic hyperuricemia from acute gout or remission of gout. The biomarkers tested in the literatures include inflammatory markers (such as IL-1 β, TNF-α) and oxidative stress pathway metabolites assayed from serum and plasma samples. However, the above-mentioned biomarkers are insufficient to distinguish the difference between two sub processes, despite each sub-process with different feature of immune response.

In order to overcome above-mentioned difficulties, this paper takes a new research method, namely functional OR-PAM, measuring oxygen saturation and microvascular images, in order to distinguish oxygen metabolism correlation with local inflammatory lesions. Based on oxygen metabolism monitoring principle, we found the recessive factors related with the spontaneous resolution of acute GA. Our measurement results not only revealed the phenomenon of ‘oxygen level reversal from hyperoxia to hypoxia’, but also show the detail of temporal variation of two sub-process. PAM has been used in quantitative assessment of topical corticosteroid application in mice skin[47], guided therapy strategies[48] especially in hypoxia condition[49]. It is worth note that we further investigated the regular value of sO_2_ in joint. Our study examined peri-articular microvasculature in the ankle joint region[50]. Our measurements revealed an average sO_2_ range of 56–72 % in these micro-vessels. Similar results have been monitored in human inflammatory arthritis study with 58 % and 65 % for arthritic joints and the normal joints, respectively[10] and < 57 % in small vessels of mouse ear[51] where tissue oxygen extraction naturally results in lower sO_2_ levels compared to large arteries. We further conducted an analysis of the tortuosity metrics related to blood vessel morphology (DM, ICM, and SOAM) for PAM images from experiments I-III. The results were plotted in Fig. S8, indicated no significant changes. The acute inflammation caused an initial enlargement of blood vessel diameter within 24 h, which then gradually decreased back to normal values. As the duration of inflammation was relatively short, there was no significant growth of new blood vessels. Therefore, the tortuosity remained relatively constant throughout the entire process.

Secondly, these findings suggest new therapeutic targets for treating conditions like gout. The results of experiment II and III have identified potential targets for developing new drugs and non-pharmacological treatment strategies. For instance, regulating the immune response during the first sub-process (1st-5th days) could help alleviate patient pain, while targeting the immune response during the second sub-process (6th-15th days) might aid in reducing the duration of the disease.

Furthermore, we found that MSU suspension dissolved earlier after external light irradiation, which is difficult to achieve by uric acid-lowering drugs in clinical application[52], [53]. However, the underlying mechanism of a quicker dissolution of MSU crystals following PBM remained unexplored. Regarding the potential role of macrophage phagocytosis, prior research has indicated that PBM is able to enhance macrophage phagocytosis. For example, PBM was shown to increase the phagocytic ability of isolated macrophages exposed to Bothrops jararacussu venom[54]. Meanwhile, PBM enhanced M1-macrophage mediated phagocytosis in the context of muscle tissue repair[55]. Regarding renal uric acid excretion analysis, in rats with local MSU injection, the uric acid level usually does not show a significant change. This is because locally injected MSU mainly causes a local inflammatory reaction in the joint area, and the MSU crystals are predominantly deposited locally, with only a small amount entering the systemic circulation, thus having little impact on overall uric acid metabolism in the rat[56]. Nevertheless, we understand that conducting renal uric acid excretion analysis could potentially offer new perspectives on the mechanism of accelerated MSU clearance by PBM. The current understanding of MSU crystal dissolution primarily centers around uric acid concentration. When serum uric acid levels decrease below the saturation level of MSU crystals, the crystals tend to dissolve gradually[15], [39]. It will be very important to explore the effect of PBM of MSU crystals in GA. While this is beyond the scope of our current work, we aim to explore this further in future research focusing on non-pharmacological treatments for gout.

## 5 Conclusion

In conclusion, our study unveiled that an inflammation-resolution process comprising a first sub-process (in 1st - 5th days) followed by a secondary sub-process (in 6th - 15th days). The first sub-process is triggered by MSU crystal deposition. Its characteristic is represented in acute GA symptoms and a hyperoxic microenvironment, as evidenced by PAM and histopathological analyses. Subsequently, the secondary sub-process is driven by second wave of immune response. Its characteristic is represented in hypoxia state, which is caused by the increased oxygen consumption of the immune response. Notably, our findings suggest that modulating the hyperoxia or hypoxia levels could serve as a promising avenue for the development of non-pharmacological therapeutic interventions targeting GA. Further exploration of these distinct inflammatory stages may offer valuable insights for the diagnosis and treatment strategies in the management of gout.

## Funding sources

This work was supported by 10.13039/501100001809National Natural Science Foundation of China (NSFC) Grant (62305396), 10.13039/501100002367Chinese Academy of Sciences Grant (Strategic Priority Research Program XDB0930000, Young Scientists in Basic Research Grant No.YSBR-104, 10.13039/501100012492Youth Innovation Promotion Association Y2023099); National Key Research and Development Program of China (2022YFE0132300, 2023YFF0715300, 2023YFC2411700, 2020YFA0908800); 10.13039/501100001809National Natural Science Foundation of China Grant (82327805); Shenzhen Science and Technology Innovation Grant (JCYJ20241202124916023, JCYJ20220818101403008, KJZD20240903101259001, KJZD20240903095714019); Key Laboratory of Biomedical Imaging Science and System, 10.13039/501100002367Chinese Academy of Sciences; State Key Laboratory of Biomedical Imaging Science and System; Guangdong Provincial Key Laboratory of Biomedical Optical Imaging (2020B121201010); Shenzhen Key Laboratory for Molecular Imaging (ZDSY20130401165820357; The Innovation Platform for Academicians of Hainan Province (YSPTZX202202).

## Authorship contribution statement

Y.T. designed the experiments, performed experiments and wrote the manuscript. M.Z. performed the PAM and light treatment experiment, analyzed data and wrote the manuscript. Z.W. built the PAM system, processed and analyzed data. J.C. and Y.R. provided conceptual input and supervised the project. Y.G. and C.L. supervised the study, provided input for the experiments and the study concept, and edited the paper.

## CRediT authorship contribution statement

**Yaguang Ren:** Software, Methodology, Investigation. **Chengbo Liu:** Writing – review & editing, Supervision, Software, Methodology, Formal analysis. **Ying Gu:** Writing – review & editing, Supervision, Investigation. **Yizhou Tan:** Writing – review & editing, Writing – original draft, Supervision, Investigation. **Min Zhang:** Writing – review & editing, Writing – original draft, Methodology, Investigation, Formal analysis, Data curation, Conceptualization. **Zhifeng Wu:** Software, Methodology, Formal analysis, Data curation. **Chen Jingqin:** Writing – original draft, Supervision, Software.

## Declaration of Competing Interest

The authors declare that they have no known competing financial interests or personal relationships that could have appeared to influence the work reported in this paper.

## Data Availability

Data will be made available on request.
