# Supplementary material for: Local oxygen concentration reversal from hyperoxia to hypoxia monitored by optical-resolution photoacoustic microscopy in inflammation-resolution process
Source: Photoacoustics. 2025 May 17;44:100730. doi: 10.1016/j.pacs.2025.100730 (PMC12162082; doi:10.1016/j.pacs.2025.100730)

**Local Oxygen Concentration Reversal from Hyperoxia to Hypoxia Monitored by Optical-Resolution Photoacoustic Microscopy in Inflammation-Resolution Process**

Yizhou Tan^a,b,1^, Min Zhang^a,c,1^, Zhifeng Wu^c^, Jingqin Chen^c^, Yaguang Ren^c^, Chengbo Liu^c,^*, Ying Gu^a,b,*^

^a^Department of Laser Medicine, the First Medical Center, Chinese PLA General Hospital, Beijing 100853, China.

^b^Laser Medicine Center, Hainan Hospital, Chinese PLA General Hospital, Sanya, 572013, China.

^c^Research Center for Biomedical Optics and Molecular Imaging, Key Laboratory of Biomedical Imaging Science and System, Shenzhen Institute of Advanced Technology, Chinese Academy of Sciences, Shenzhen, 518055, China.

* Corresponding authors.

*E-mail addresses:* guyinglaser301@163.com (Y. Gu), cb.liu@siat.ac.cn (C. Liu)

^1^These authors contributed equally to this work.


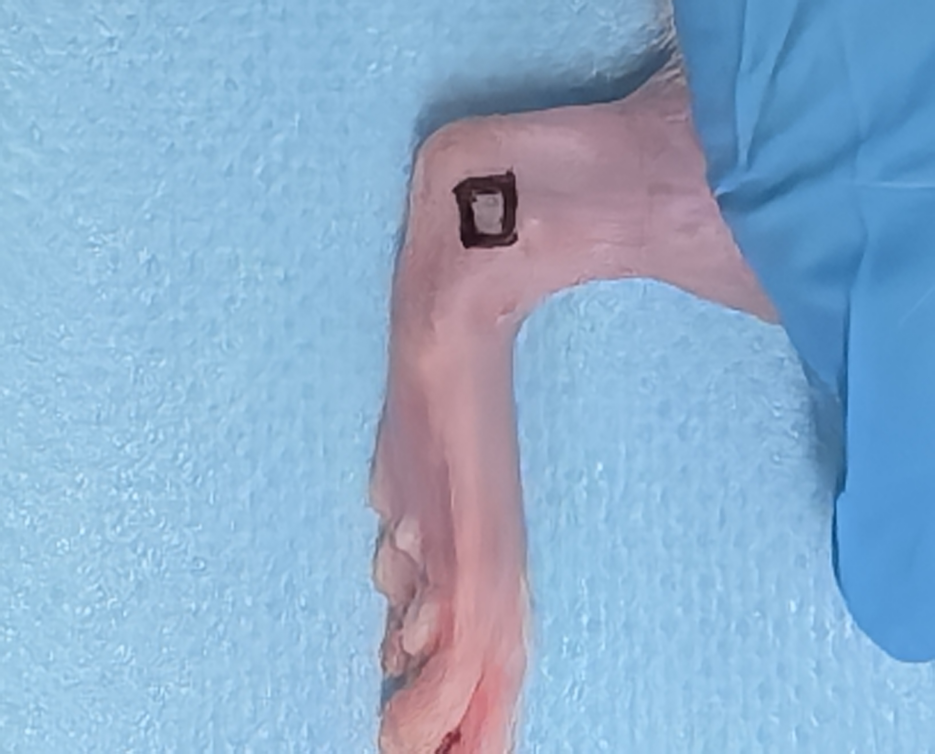

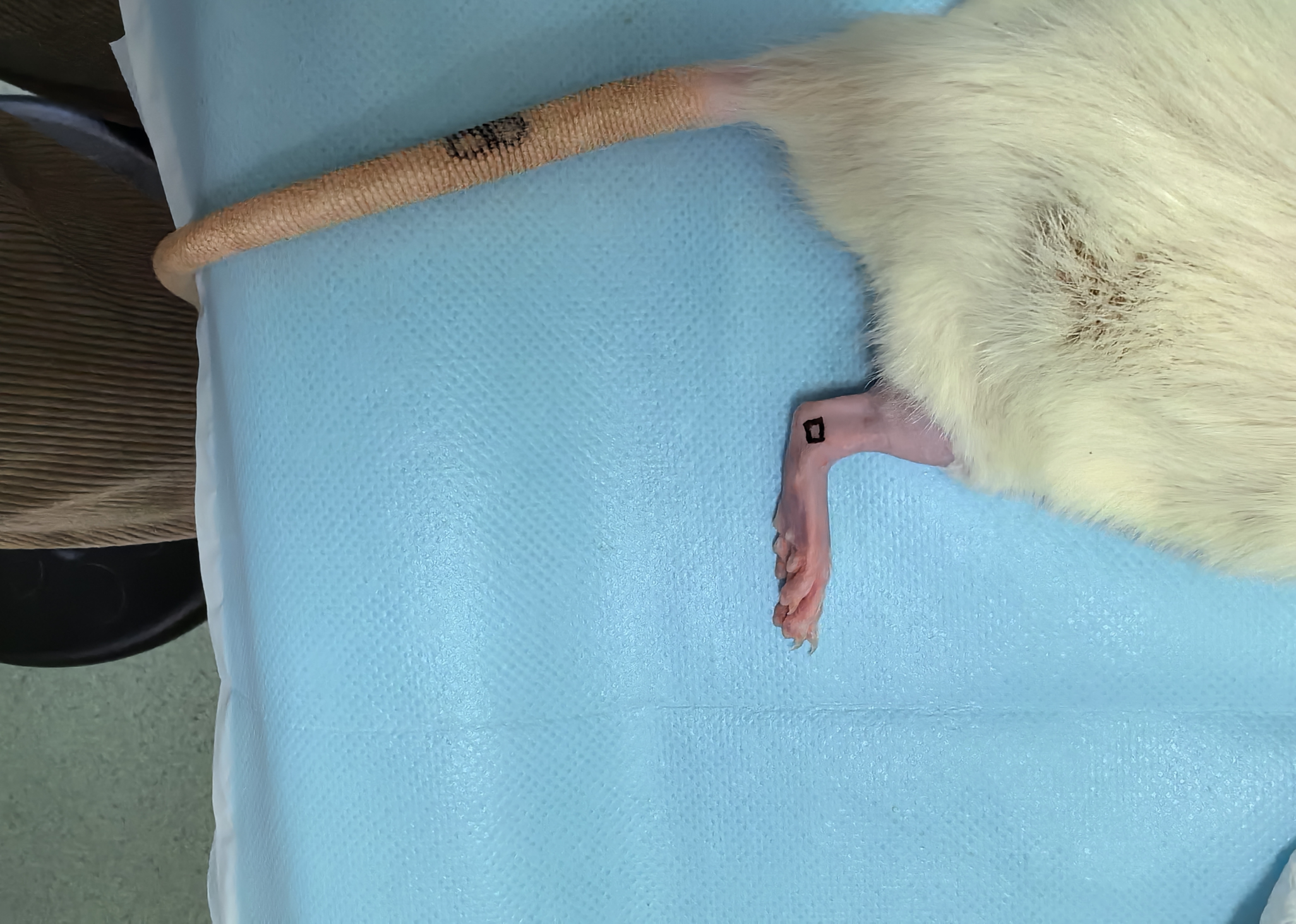


**Fig. S1.** Marker on ankle joint for PAM imaging.


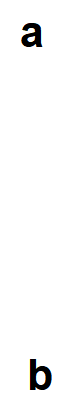

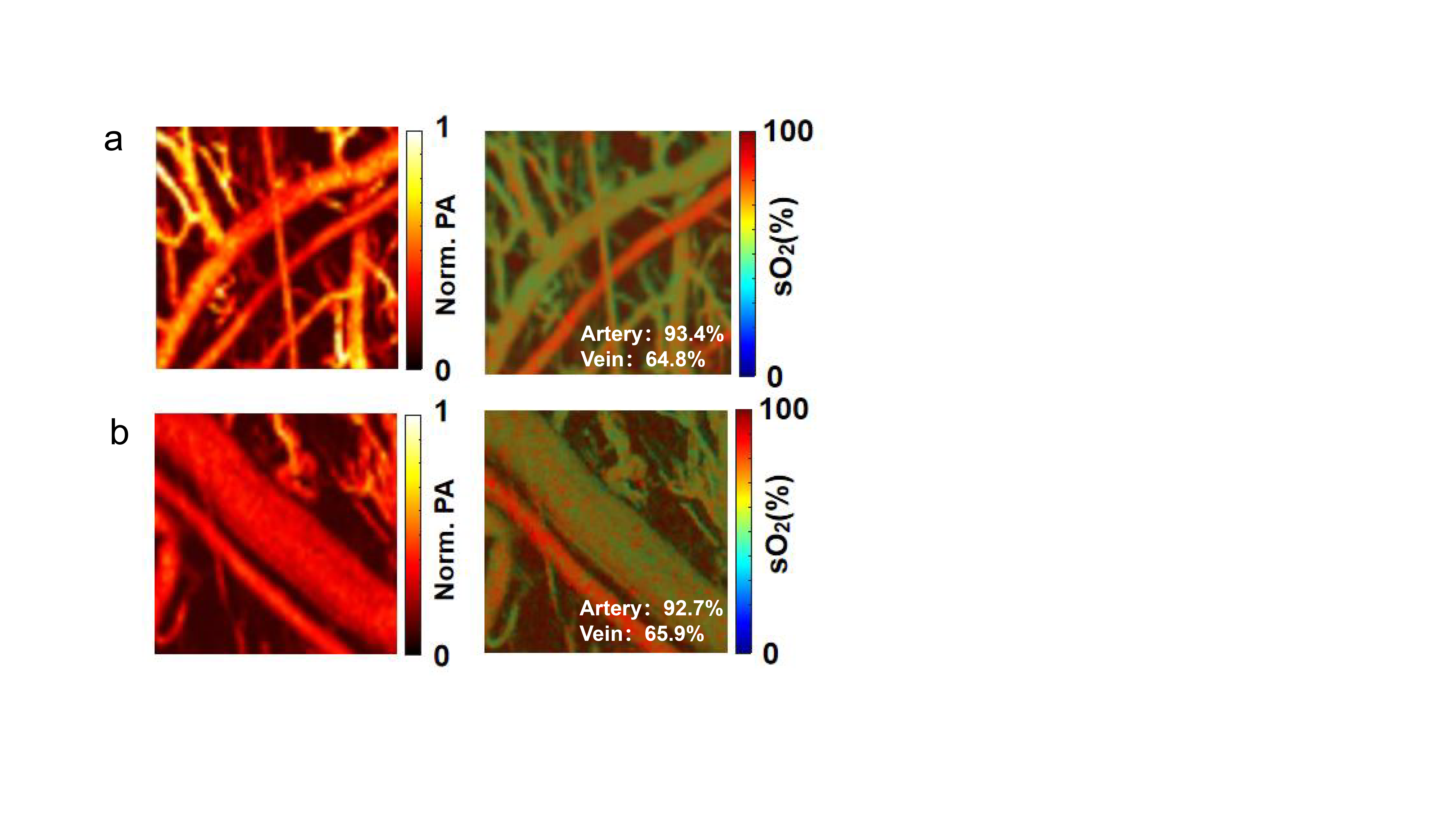


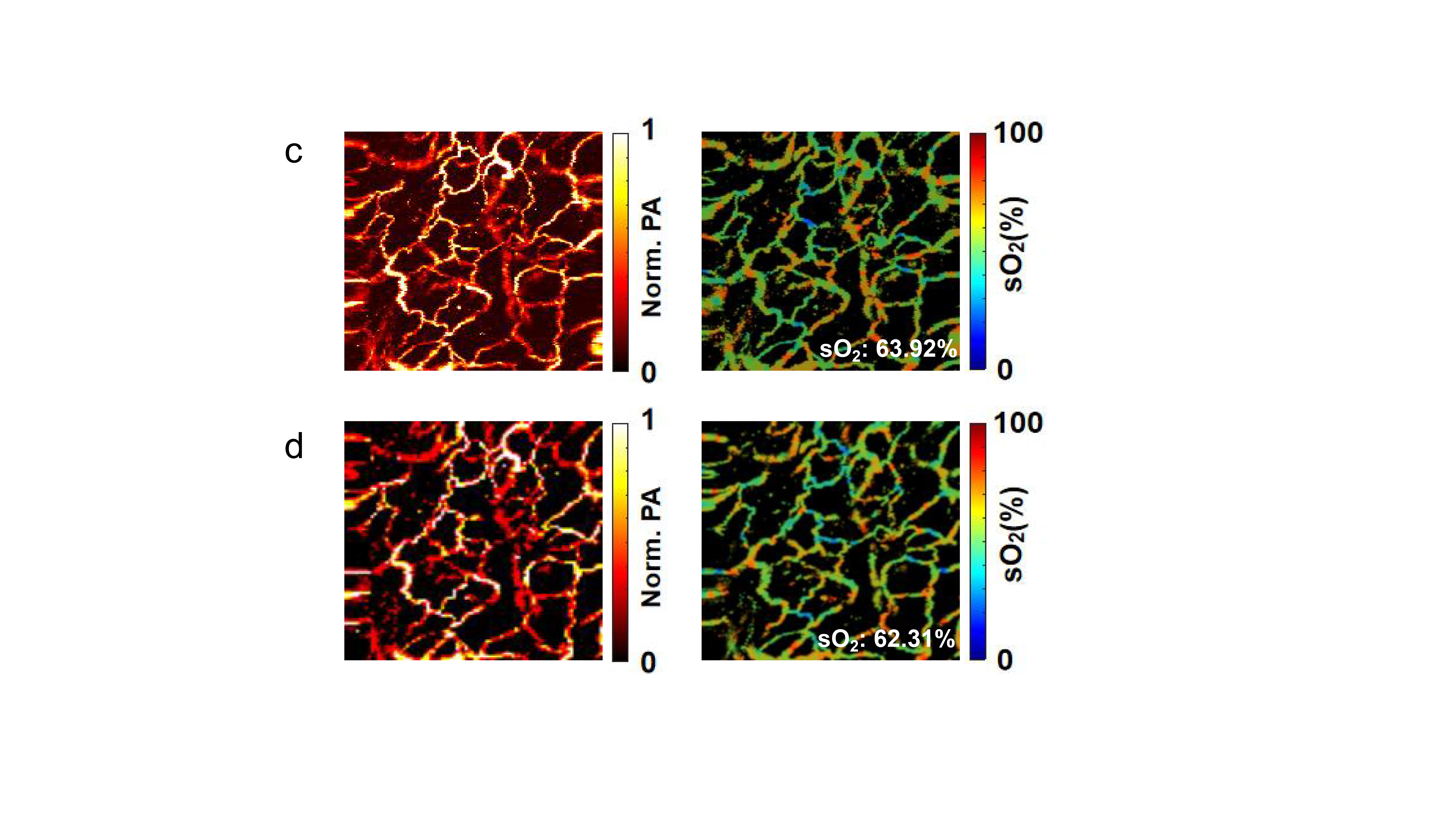


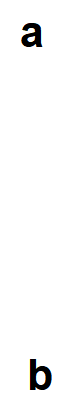


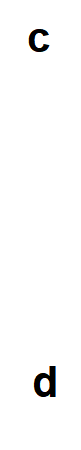


**Fig. S2.** Reliability of the system for accurate oxygen saturation imaging. Left column: PA images of blood vessels. Right column: sO_2_ images of blood vessels. (a, b) PA image at different positions on the back of nude mice. (c, d) Two repeat PA scans at the same position of the rat ankle.


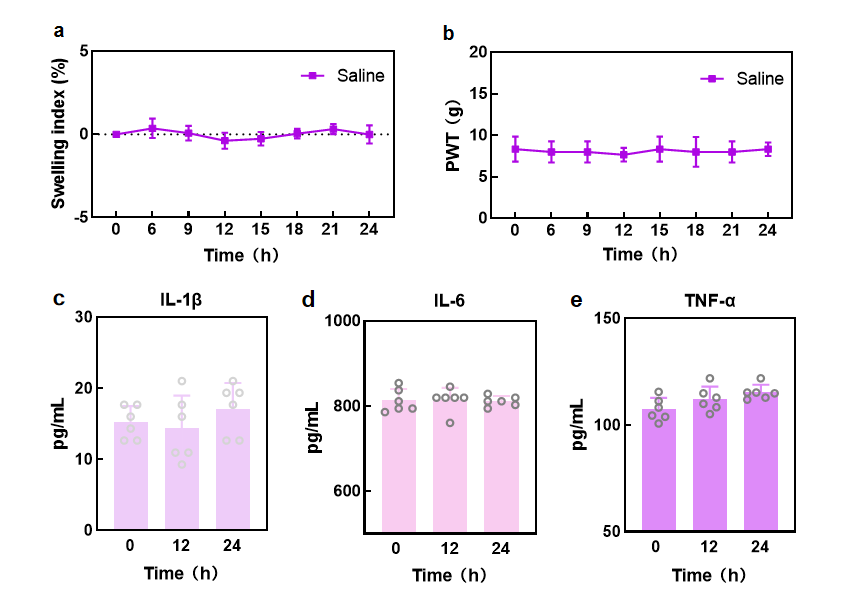


**Fig. S3.** **The changes of rats within 24 hours after injection of saline solvent were observed.** (a) Ankle swelling index analysis of control group rats in 24 hours. (b) PWT value of von Frey filament test of control group in 24 hours. (c-e) Analysis of serum inflammatory cytokines: IL-1β, IL-6, TNF-α before the injection, 12 hours and 24 hours after the injection. Animal number n = 6, mean ± SD.


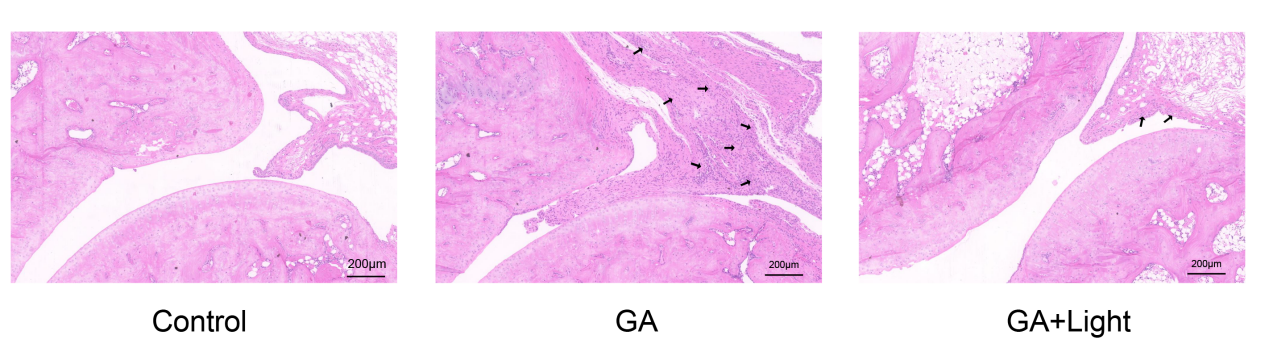


**Fig. S4.** Histopathological results of synovium from control group, synovium from GA group and synovium from GA+Light group. Inflammatory cells were seldomly found in the healthy joint tissue in control group. However, synovial tissue in GA group was hyperplasia with a large number of inflammatory cells infiltrated into synovial tissue and angiogenesis observed. Compared with GA group, the infiltration of inflammatory cells into synovial tissue in GA+Light group was significantly less. Black arrows indicated inflammatory cells.


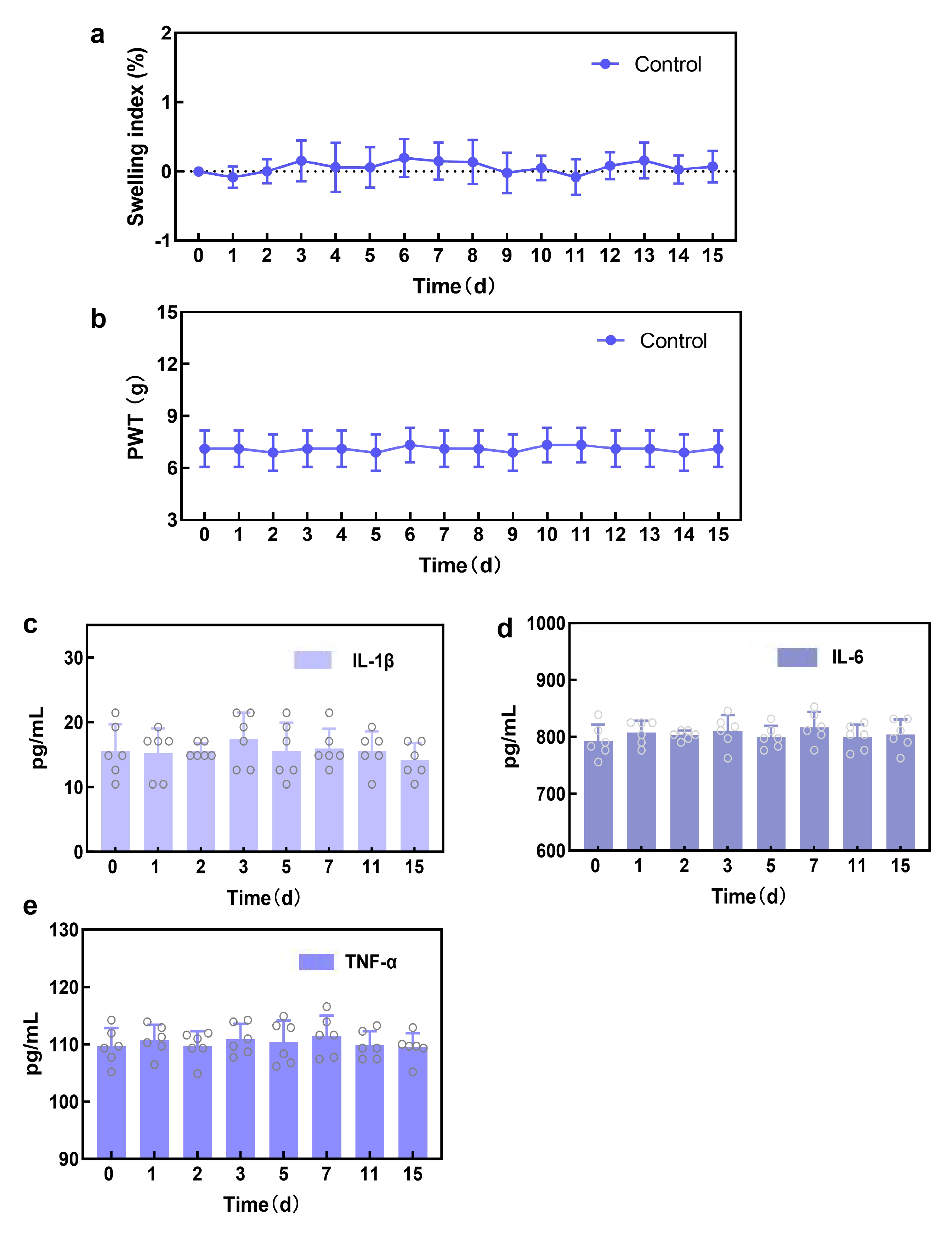


**Fig. S5.** **The changes of rats within 15 days after injection of normal saline were observed. (a)** Ankle swelling index analysis of control group rats in 15 days. **(b)** PWT value of von Frey filament test of control group in 15 days. **(c-e)** Analysis of serum inflammatory cytokines: IL-1β, IL-6, TNF-α at time. Animal number n = 6, mean ± SD.


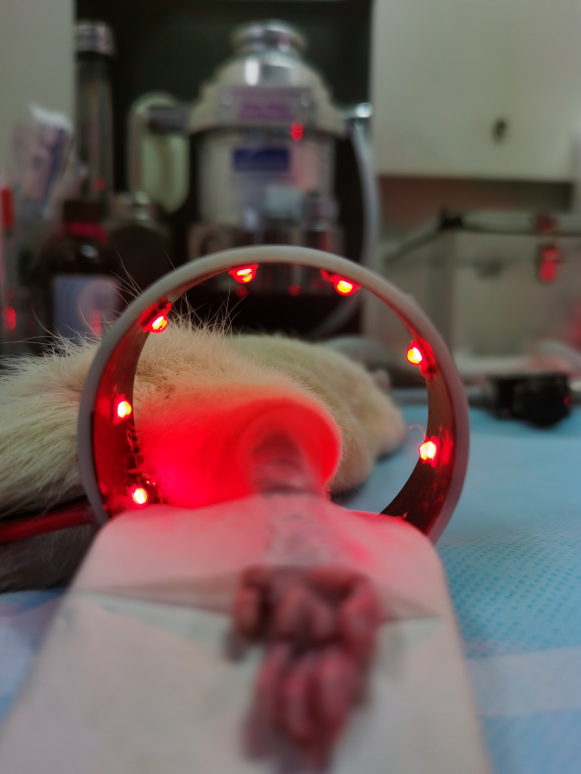


**Fig. S6.** An external light irradiation device for treating GA joints.


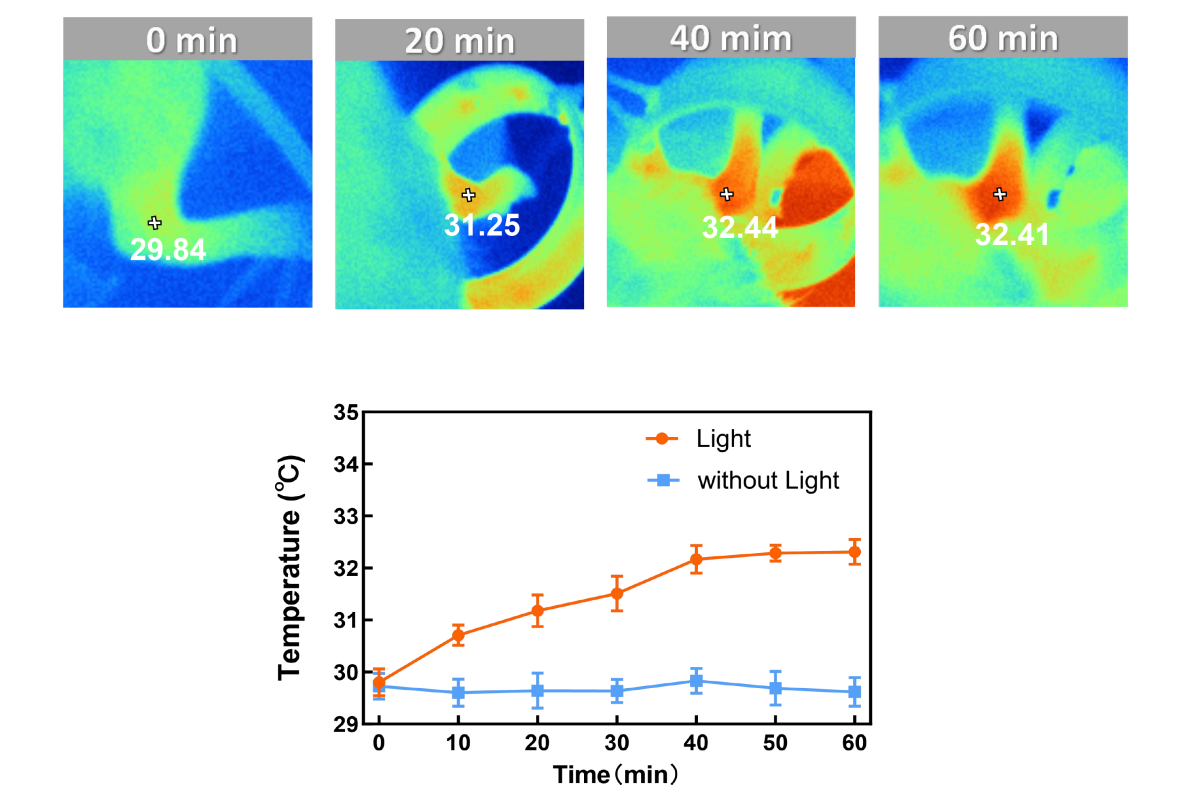


**Fig. S7.** Temperature of joint surface during the external light irradiation. Animal number n = 6, mean ± SD.


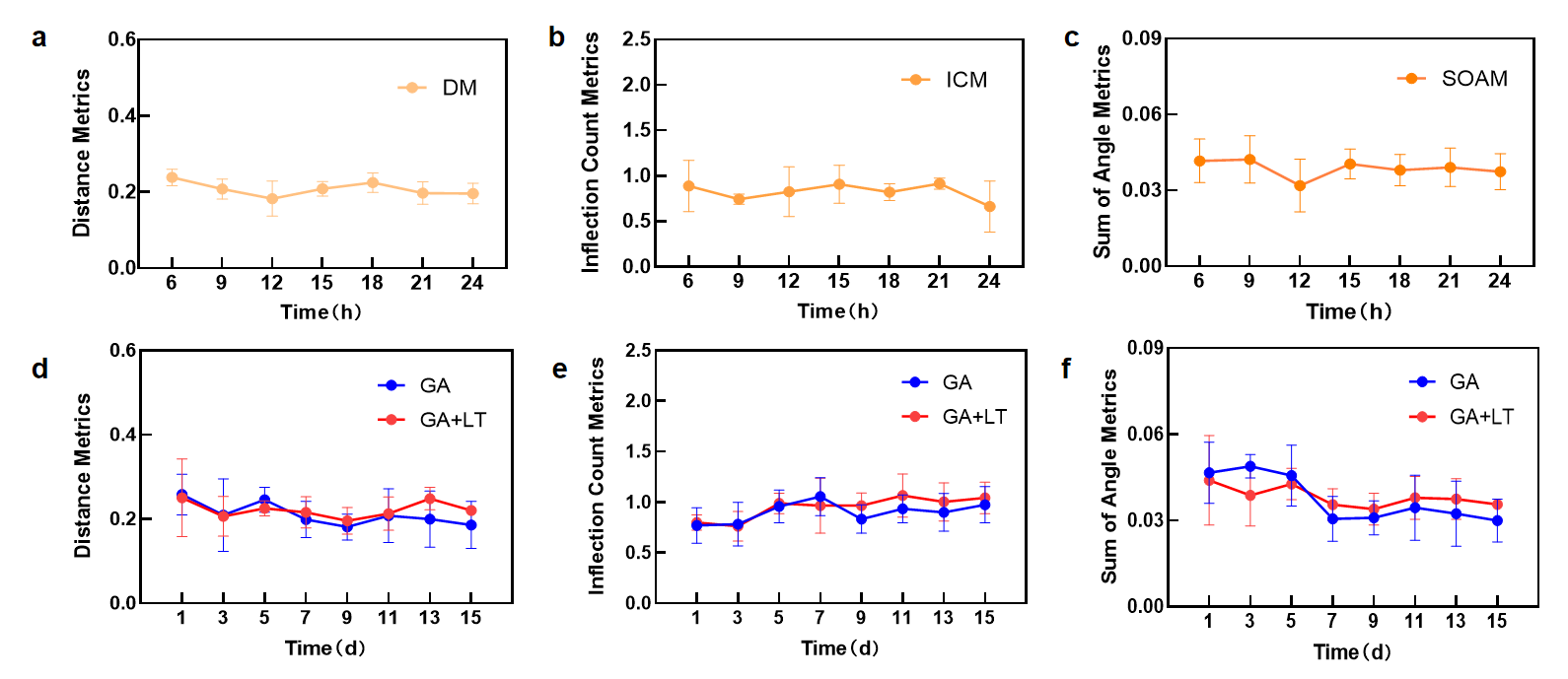


**Fig. S8. Observation of vascular tortuosity in SD rats.** (a) Averaged the distance metric of blood vessels in GA ankles within 24h. (b) Averaged the inflection count metric of blood vessels in GA ankles within 24h. (c) Averaged the sum-of-angles metric of blood vessels in GA ankles within 24h. (d) Comparison of the distance metric between GA and GA+Light group. (e) Comparison of the inflection count metric between GA and GA+Light group. (f) Comparison of the sum-of-angles metric between GA and GA+Light group. Animal number n = 6, mean ± SD.

**Tab. S1.** Numerical changes and statistical results of swelling index. Animal number n = 6, mean ± SD.


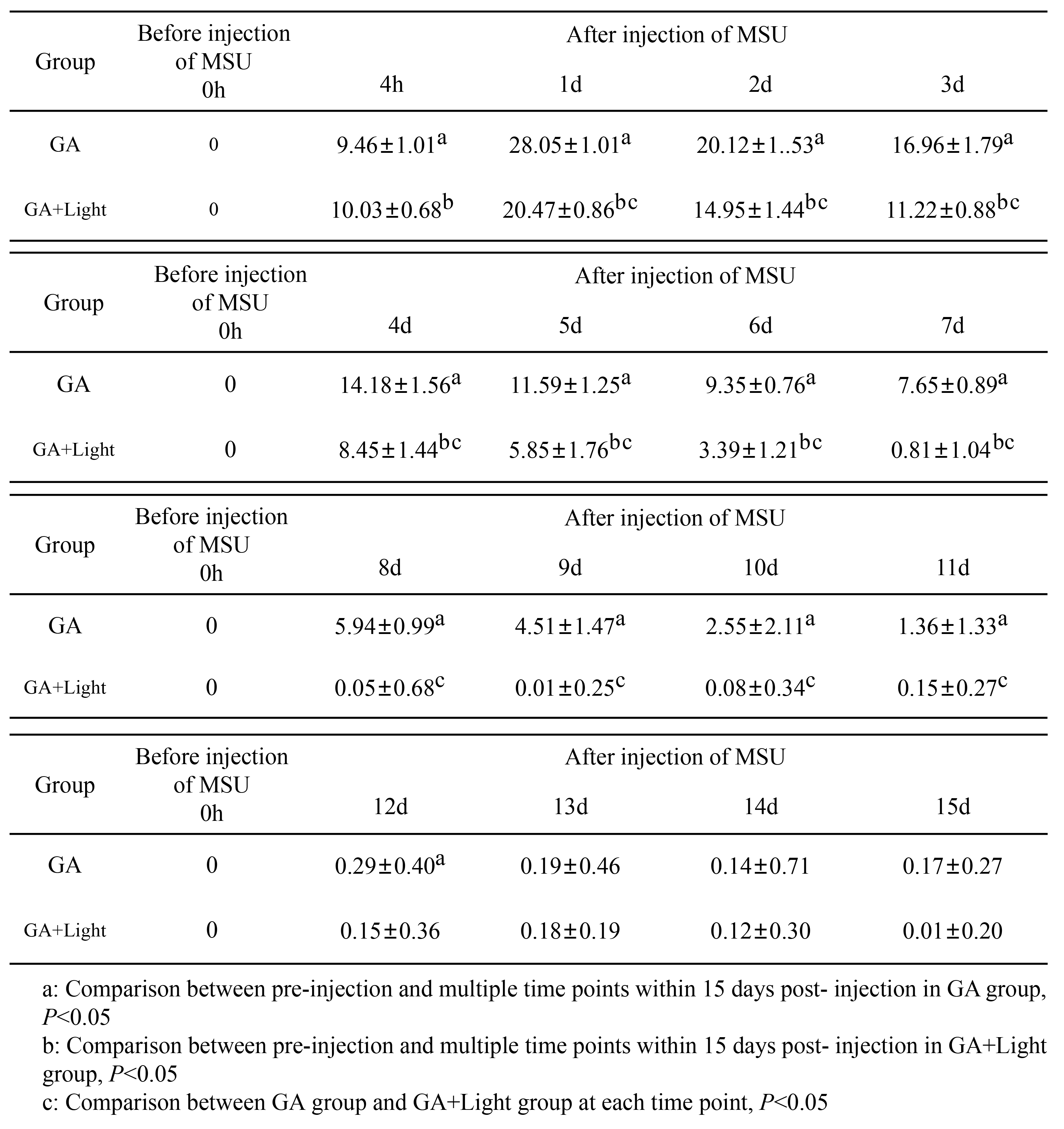


**Tab. S2.** Numerical changes and statistical results of PWT value from von Frey test. Animal number n = 6, mean ± SD.


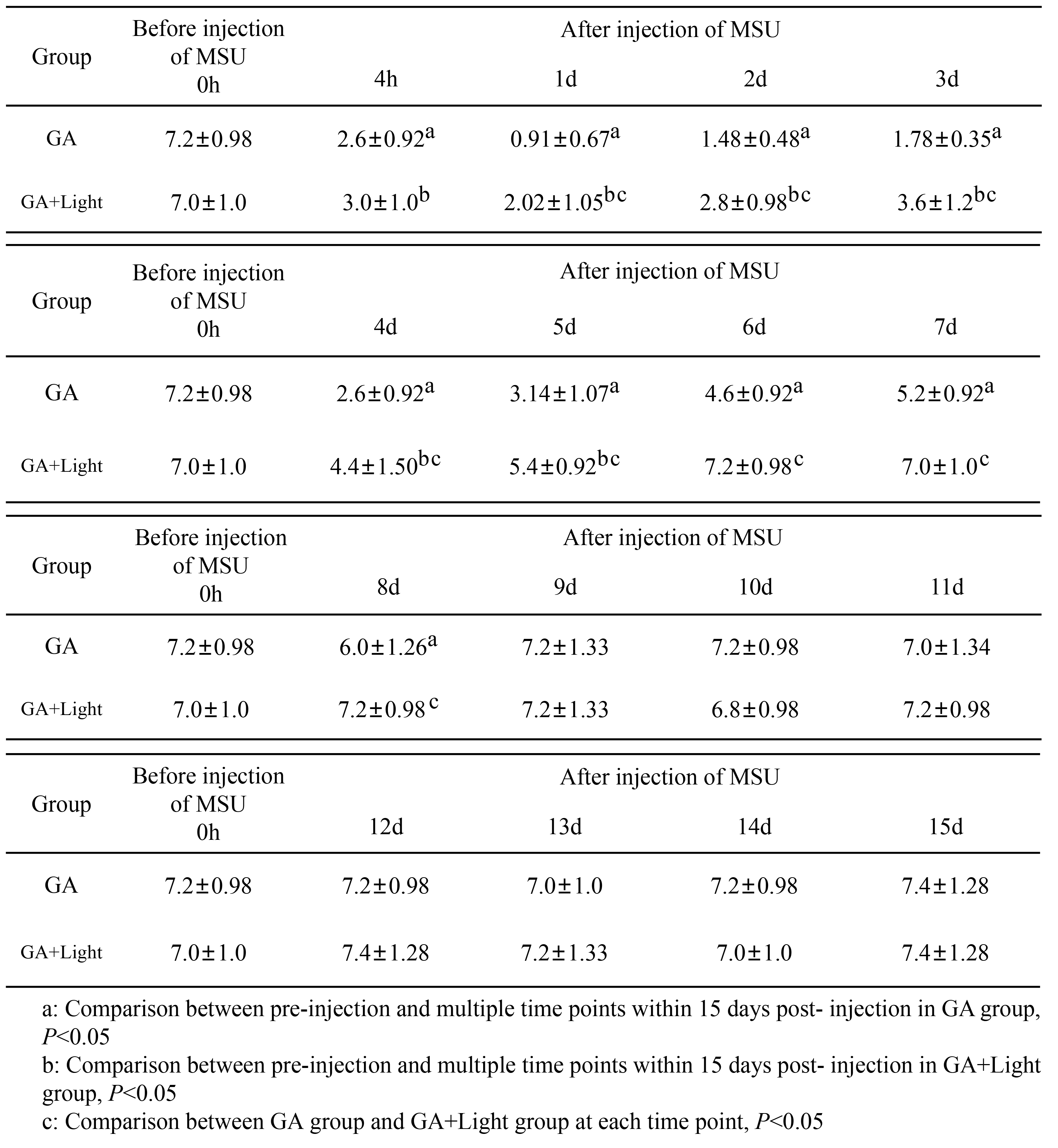


**Tab. S3.** Numerical changes and statistical results of vascular diameter. Animal number n = 6, mean ± SD.


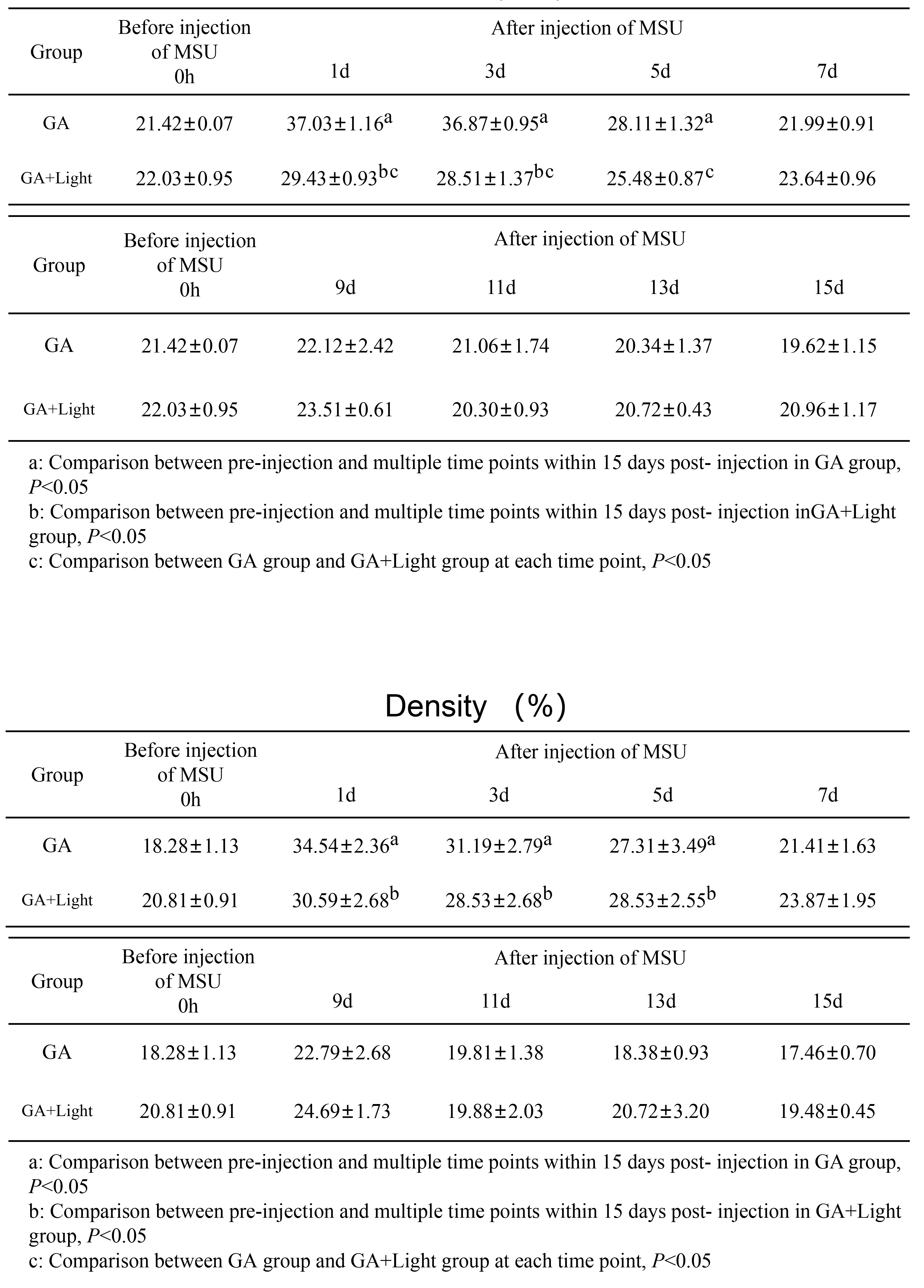


**Tab. S4.** Numerical changes and statistical results of vascular density. Animal number n = 6, mean ± SD.


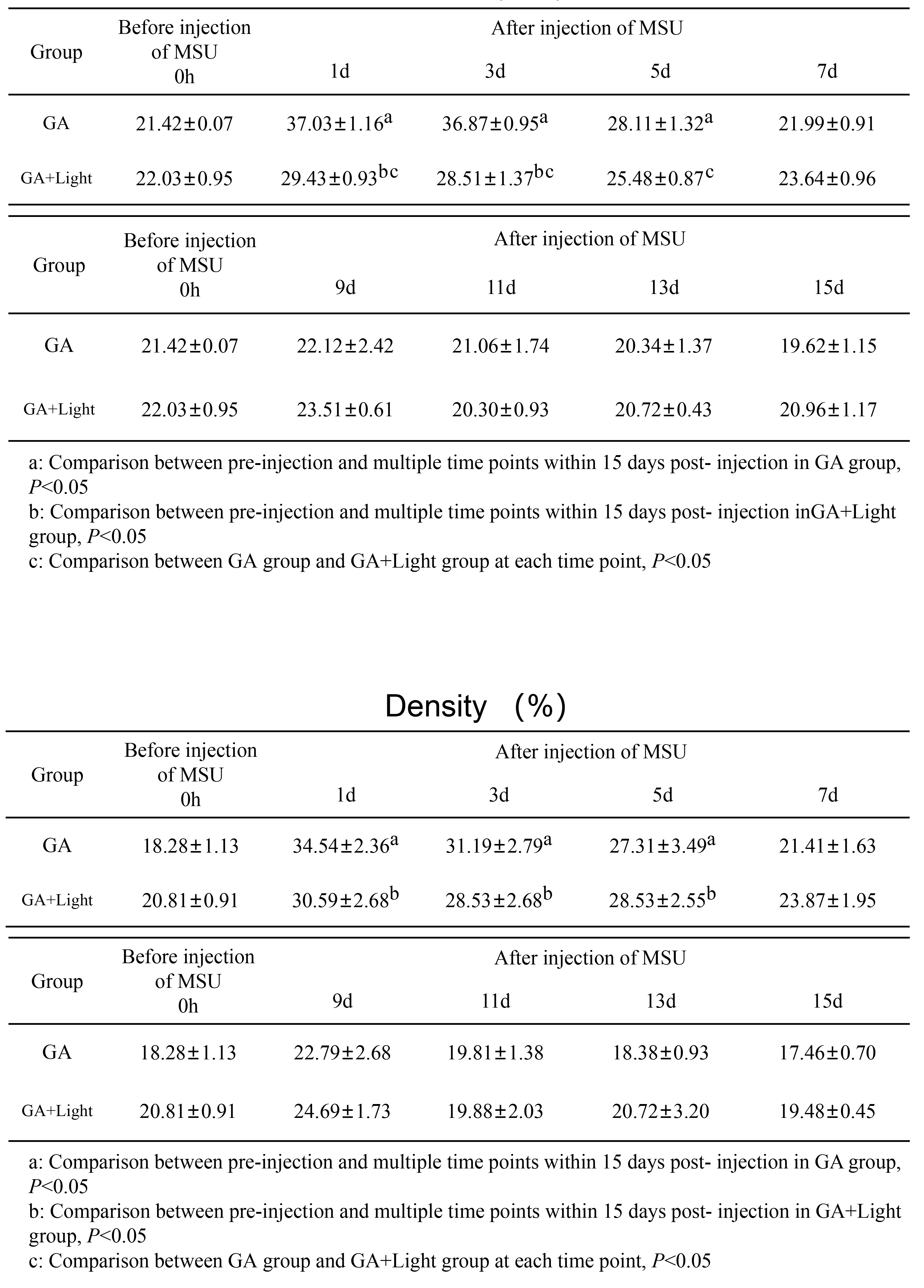


**Tab. S5.** Numerical changes and statistical results of blood sO_2._ Animal number n = 6, mean ± SD.
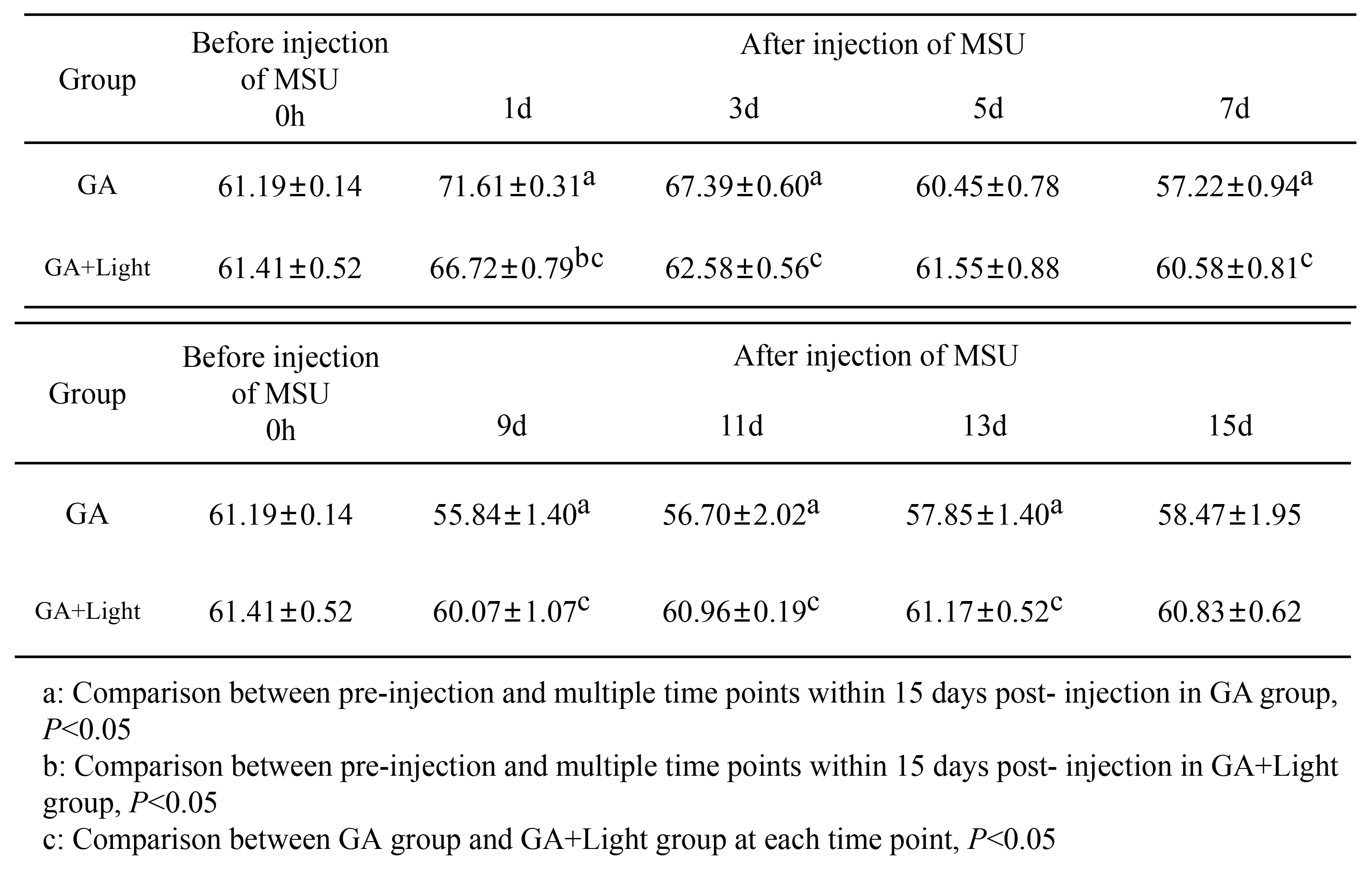


**Tab. S6.** Numerical changes and statistical results of MSU area. Animal number n = 6, mean ± SD.


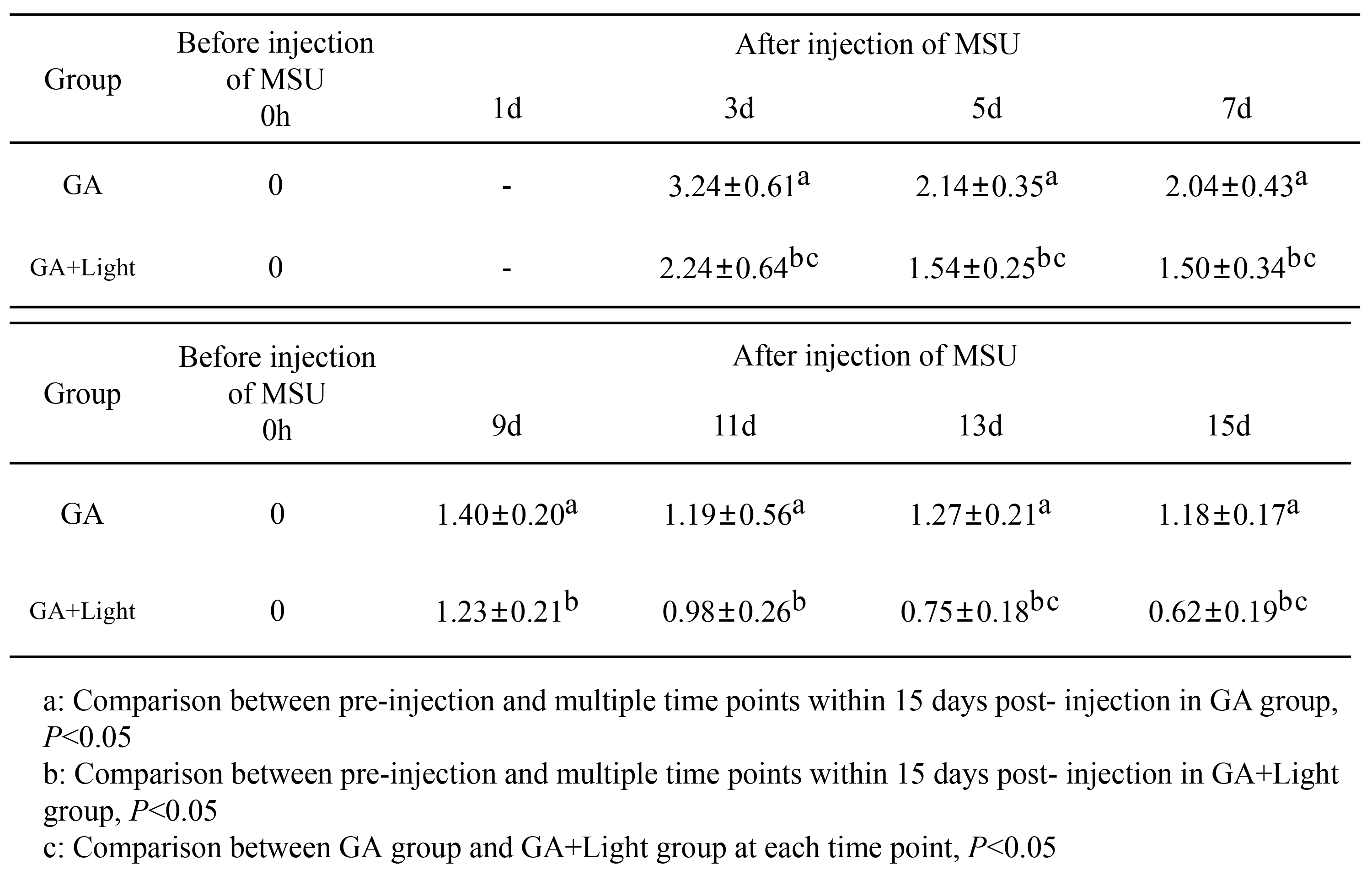


**Tab. S7.** Numerical changes and statistical results of serum inflammatory cytokines: IL-1β, IL-6, TNF-α. Animal number n = 6, mean ± SD.


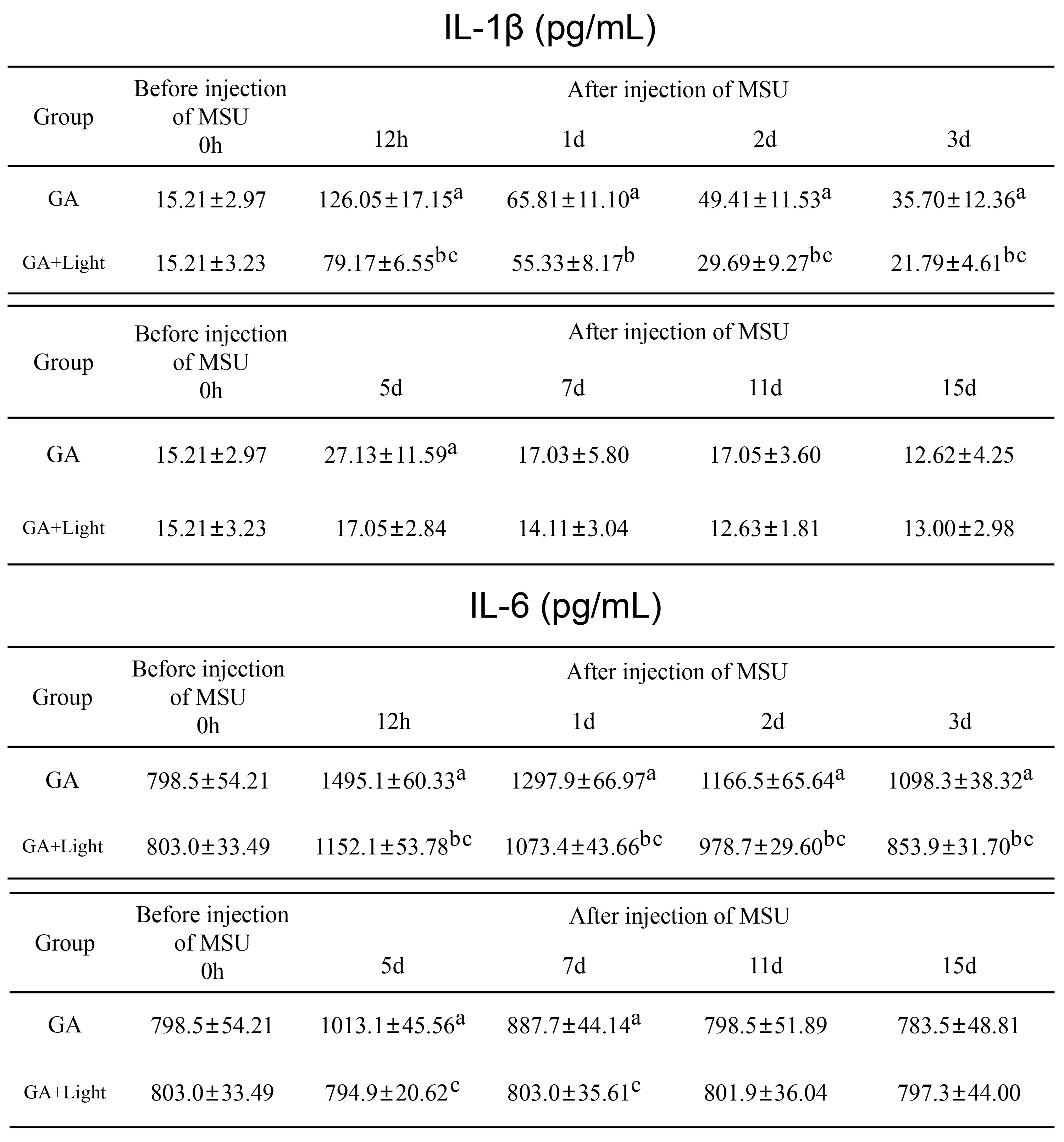


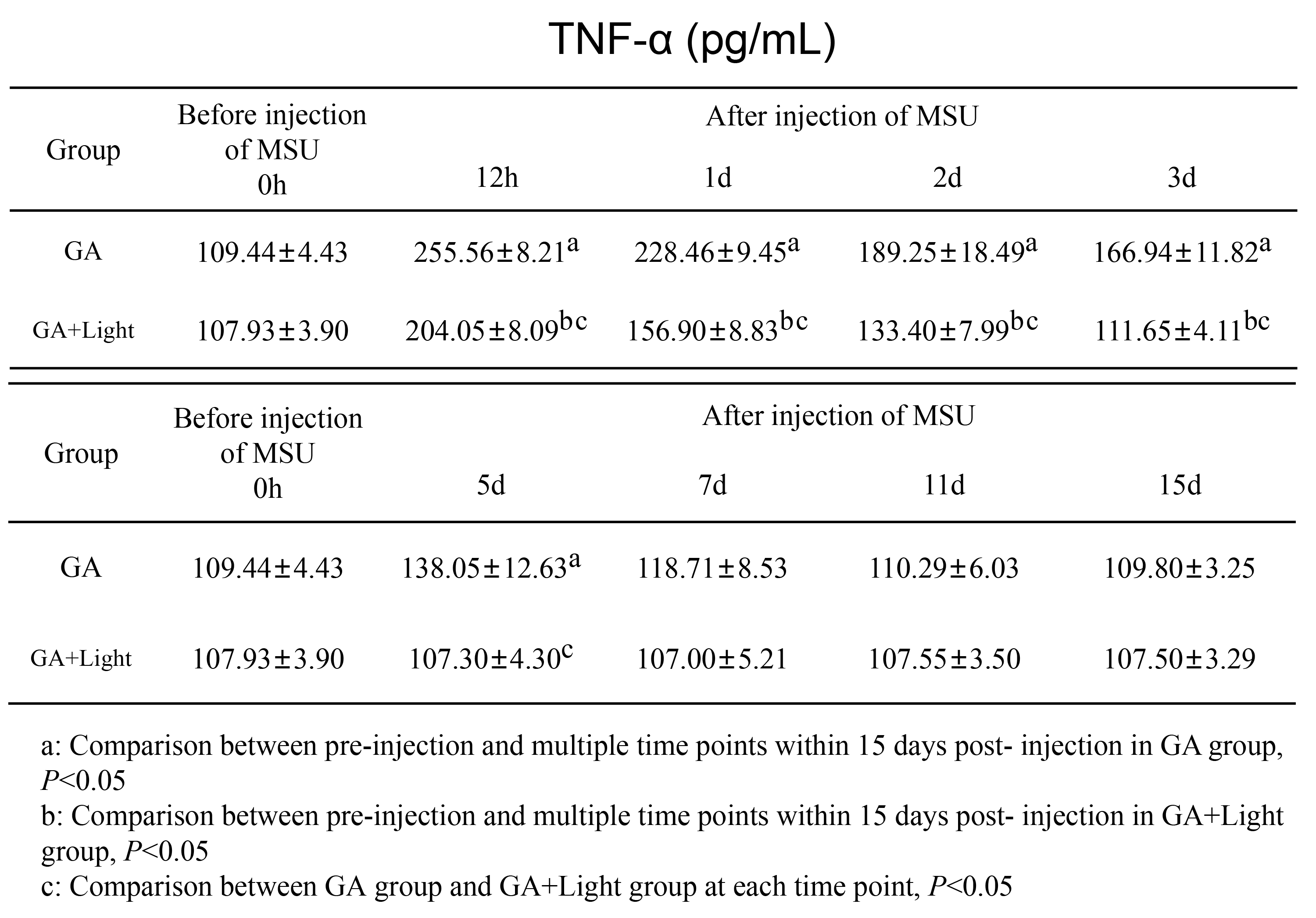

Supplement: Supplementary file 1 — Supplementary material [file mmc1.docx]
